# Associations between basic physiological observations recorded pre-thrombectomy and functional outcome: a systematic review and meta-analysis

**DOI:** 10.3389/fstro.2023.1283312

**Published:** 2023-10-19

**Authors:** Hannah A. Lumley, Lisa Shaw, Julia Morris, Abi Alton, Phil White, Gary A. Ford, Martin James, Christopher Price

**Affiliations:** ^1^Population Health Sciences Institute, Newcastle University, Newcastle upon Tyne, United Kingdom; ^2^Translational and Clinical Research Institute, Newcastle University, Newcastle upon Tyne, United Kingdom; ^3^Newcastle upon Tyne Hospitals NHS Foundation Trust, Newcastle upon Tyne, United Kingdom; ^4^Oxford University Hospitals NHS Foundation Trust and Medical Sciences Division, University of Oxford, Oxford, United Kingdom; ^5^Peninsula Applied Research Collaboration (PenARC), University of Exeter, Exeter, United Kingdom

**Keywords:** thrombectomy, outcome, prognosis, modified Rankin, physiological observations

## Abstract

**Introduction:**

Mechanical thrombectomy results in more favourable functional outcomes for patients with acute large vessel occlusion (LVO) stroke. Key clinical determinants of thrombectomy outcome include symptom severity, age and time from onset to treatment, but associations have also been reported with baseline physiological observations including systolic/diastolic blood pressure (SBP/DBP), blood/serum glucose, atrial fibrillation and conscious level. As these items are routinely available during initial emergency assessment, they might help to inform early prehospital and hospital triage decisions if evidence consistently shows associations with post-thrombectomy outcome. We undertook a meta-analysis of studies reporting pre-thrombectomy physiological observations and functional outcome.

**Method:**

PRISMA guidelines were followed to search electronic bibliographies, select articles and extract data. Medline, PubMed, Cochrane HTA, Cochrane Central and Embase were searched. Included articles were observational or interventional thrombectomy studies published between 01/08/2004-19/04/2023 reporting 3-month modified Rankin Scale, split as favourable (0–2) and unfavourable (3–6). A modified version of the Quality in Prognostic Studies (QUIPS) tool was used to assess risk of bias. RevMan 5 was used to calculate Inverse Variance with Weighted Mean Differences (WMD) and Mantel-Haenszel Odds Ratios (OR) for continuous and categorical factors respectively.

**Results:**

Thirty seven studies were eligible from 8,687 records. Significant associations were found between unfavourable outcome and higher blood/serum glucose as a continuous (WMD = 1.34 mmol/l (95%CI 0.97 to 1.72); 19 studies; *n* = 3122) and categorical (OR = 2.44 (95%CI 1.9 to 3.14) variable; 6 studies; *n* = 5481), higher SBP (WMD = 2.98 mmHg (95%CI 0.86 to 5.11); 16 studies; *n* = 4,400), atrial fibrillation (OR = 1.48 (95%CI 1.08 to 2.03); 3 studies; *n* = 736), and lower Glasgow Coma Scale (WMD = −2.72 (95%CI −4.01 to −1.44); 2 studies; *n* = 99). No association was found with DBP (WMD = 0.36 mmHg (95%CI −0.76 to 1.49); 13 studies; *n* = 3,614).

**Conclusion:**

Basic physiological observations might assist early triage decisions for thrombectomy and could be used in combination with other information to avoid futile treatment and ambulance transfers. It is important to acknowledge that data were only from thrombectomy treated patients in hospital settings and it cannot be assumed that the predictors identified are independent or that modification can change outcome. Further work is needed to establish the optimal combination of prognostic factors for clinical care decisions.

## Background

Stroke is a medical emergency requiring urgent assessment and treatment (National Institute for Health and Care Excellence, 2019; Zhou et al., 2022). Selected patients with ischaemic stroke due to large vessel occlusion (LVO) should receive mechanical thrombectomy, a highly effective procedure with a number needed to treat (NNT) of 3 patients to reduce disability by one point on the modified Rankin scale (mRS) (Goyal et al., 2016). The main determinants of thrombectomy outcomes are age, time from onset to treatment, baseline stroke severity and radiological variables such as ischaemic core volume and collateral blood supply (Sarraj et al., 2013; Goyal et al., 2016; Evans et al., 2017; Mokin et al., 2017, 2019; Nogueira et al., 2018; Cappellari et al., 2020; Diestro et al., 2020; Ramos et al., 2020; Wang and Zhou, 2020; Kremers et al., 2021; Venema et al., 2021). However, an increasing number of reports also describe potentially valuable relationships with basic clinical information, including physiological variables (e.g., blood pressure, glucose, oxygen saturation, temperature), and futile recanalisation (i.e., reperfusion with no clinical improvement) (Zhou et al., 2022). These factors are potentially of interest because they are routinely collected during initial clinical assessment by non-specialist emergency responders such as paramedics (Quinn et al., 2022) prior to specialist review and brain imaging, and could enhance early selection of patients for direct admission to a thrombectomy centre when this is not the closest hospital. They may also be useful in hospital to modify treatment decisions if there is uncertainty about the value of thrombectomy for patients at the margins of standard criteria [e.g., with mild stroke severity towards the end of treatment time windows (National Institute for Health and Care Excellence, 2019; Sentinel Stroke National Audit Programme, 2023)]. However, the evidence describing the relationships between physiological observations and treatment outcome is mixed and of variable quality (Nie et al., 2023).

Previous reviews have considered the prognostic relationship between specific physiological observations and thrombectomy outcome. For example, a systematic review of blood pressure showed that patients with hypertension had significantly higher odds of an unfavourable (mRS 3–6) functional outcome (odds ratio 0.70; 95% CI 0.57–0.85) (Yuan et al., 2020). However, this review did not differentiate between a previous history of high blood pressure and actual pre-thrombectomy blood pressure values. It is uncertain whether these have equivalent effects. Another review found that higher blood glucose was significantly associated with lower odds of a favourable (mRS 0–2) functional outcome (odds ratio 0.92, 95% CI 0.09–0.95) (Chamorro et al., 2019), but only included data from seven randomised trials comparing thrombectomy with standard care between 2010 and 2017.

As there have been significant advances in thrombectomy and patient selection over the last six years, an up-to-date comprehensive review is required to consider the relationship between outcomes and pre-treatment physiological observations, including data from real-world populations as well as clinical trials with strict inclusion criteria. We aimed to synthesise all published evidence in a meta-analysis describing the association between a clinically important functional outcome post-thrombectomy (3-month mRS split as favourable (0–2) and unfavourable (3–6) functional outcome) and individual basic physiological observations, which could be collected pre-thrombectomy without specialist training.

## Methodology

The review was conducted in accordance with PRISMA (Transparent Reporting of Systematic Reviews and Meta-Analyses) 2020 guidance from EQUATOR (Page et al., 2021) using a PICOTS research question format (Riley et al., 2019).

### Search strategy

The following electronic databases were searched using a combination of MeSH terms and keywords: MEDLINE, PubMed, Cochrane HTA, Cochrane Central and EMBASE. The search strategy was developed in collaboration with an information scientist (see Supplementary Table S1). Searches only included research published after FDA approval of the first intra-arterial thrombectomy device for acute ischaemic stroke (01/08/2004). The most recent search was on 19/04/2023. No restrictions were placed on country of origin, although searches were restricted to papers with abstracts published in the English language. Only completed studies were included; study protocols were excluded. Literature reviews and individual case studies were excluded.

### Study selection

Observational, interventional, prospective or retrospective research studies were selected for inclusion if they reported data describing the relationship between standard physiological observations collected pre-treatment and post-thrombectomy outcome reported as favourable (0–2) vs. unfavourable (3-6) mRS at 3 months. The search was not limited to this outcome to prevent excluding relevant papers with an alternative description of the same outcome.

Inclusion criteria were defined using a PICOTS approach (Table 1) and incorporated into a Study Selection Form (Supplementary Table S2). Studies were excluded if interventionists primarily used first generation devices which are no longer in routine use, e.g., MERCI (NICE, 2018). Patients were only included if they were treated with stentrievers, aspiration or a combined approach. Duplicate studies including the same patients and case reports were excluded. It was necessary for studies to present data which could be combined into a meta-analysis: i.e., group proportions and/or comparable/convertible measurements were reported.

**Table 1:** Meta-analysis of physiological observations as thrombectomy prognostic factors PICOTS components.

Population	All patients with ischaemic stroke due to large vessel occlusion who underwent mechanical thrombectomy. Pre-selected patient populations were excluded (e.g., specific gender only). Case studies (*N =* 1) were excluded
Index factor	Physiological observations routinely collected by or available to non-specialists during initial clinical assessment. Studies reporting information only available after specialist assessment, e.g., radiological characteristics, without separately reporting physiological observations were excluded. Factors had to be clearly defined and there had to be >1 study per factor
Comparator	N/A. There was no comparison of clinical care
Outcomes (Health outcomes of the targeted individual)	Favourable (0-2) vs. unfavourable (3-6) modified Rankin Score (mRS)
Timing	Index factors routinely available and measured before thrombectomy treatment. Outcome measured at 3 months post-treatment
Setting	Thrombectomy provided in any clinical care setting using licenced devices still in routine use at the time of the literature search

One reviewer (HL) independently assessed initial eligibility of the titles and abstracts retrieved via the search strategy using the literature review screening software, “Rayyan” (Mourad et al., 2016). The same reviewer further assessed eligibility of the retained full text studies. Any uncertainties were queried with another (medically qualified) member of the review team (CP/LS).

### Data extraction

Three reviewers (HL, JM and AA) each independently extracted information from one third of the retained full text articles using a standard data extraction form (Supplementary Table S3) in Microsoft Excel. Extracted data included: Basic Study Information (e.g., Author, Year and Title), Methodological Information (e.g., Design, Criteria, Clinical Factor(s), Outcome measures, Timing; Clinical Treatment Context) and Study Results (e.g., Sample size, Predictive value; Statistics relevant to meta-analysis). All reviewers cross-checked 5% of their allocation to confirm agreement, and all extracted data were reconfirmed by HL. Any uncertainties regarding data extraction at any stage were discussed amongst the three reviewers and adjudicated by a further member of the review team when necessary. Authors were not contacted regarding missing data, and all data extracted reflected that contained in the text, tables, figures and/or Supplementary material of published reports. Studies in other languages included for having English abstracts were translated by Google Translate.

### Quality assessment

Risk of Bias (RoB) was evaluated by a modified version of the Quality in Prognostic Studies (QUIPS) tool (Riley et al., 2019) (Supplementary Table S4). The tool was simplified to align with the review aims and the anticipated variable nature of the literature. Assessment domains include: (1) Study participation, (2) Study attrition, (3) Prognostic factor measurement, (4) Outcome measure, (5) Confounding factors, (6) Statistics and reporting. Studies were categorised into tertiles as having low (score of 10.5–14/14), moderate (4–10/14), or high (0–3.5/14) risk of bias.

### Data synthesis

No transformations were performed on variable data apart from the conversion of glucose values reported in mg/dL into mmol/L for direct comparison. Binary glucose thresholds are reported in their original units in Supplementary Tables S6–S10 but following transformation it was possible to combine studies using a threshold of 7.8 mmol/L (140 mg/dL). Some categorical thresholds were negligibly different (<7.8 mmol/L vs. >/ = 7.8 mmol and </ = 7.8 mmol/L vs. >7.8 mmol/L) so were merged. Although serum and blood glucose values are reported to differ by ~1% due to the presence and absence of red blood cells, data were pooled without correction because this is a very small effect which would be very unlikely to influence the difference between favourable and unfavourable outcomes. Glasgow Coma Scale (GCS) scores could be included if reported as continuous/parametric data, although it was anticipated that this would limit study eligibility as it is typically an ordinal scale. Papers reporting AF were only included if this had been confirmed by ECG during the assessment for thrombectomy treatment, which ensured that its presence was temporally related to the procedure and detection was consistent across studies, thereby reducing heterogeneity in the analysis.

Review Manager 5 (Review Manager 2020) (Nordic Cochrane Centre, 2020; Jonathan et al., 2021) was used to produce forest plots assessing associations between prognostic factors and unfavourable (mRS 3–6) 3-month functional outcomes. For continuous factors, Inverse Variance was used as the statistical method and Weighted Mean Difference (WMD) was used as the effect measure as outcomes were measured in the same way across studies. For binary factors, the Mantel-Haenszel method was used with Odds Ratios (with 95% CIs) as the effect measure. Risk of bias (modified QUIPS) scores were added to the forest plots but were not adjusted for in the analysis.

We assessed the extent of heterogeneity between trial results for each factor using the *I*^2^ statistic, which measures the percentage of the variability in effect estimates attributable to heterogeneity rather than sampling error, in conjunction with Tau^2^ for measuring variance between studies (Higgins, 2020). We considered *I*^2^ >50% and Tau^2^ >0.5 as substantial heterogeneity indicating that studies do not share a common effect. Random effects models were used regardless of heterogeneity because it was assumed that there would always be differences between patient populations and clinical care in different study settings. Fixed effects models were used if a factor had fewer than 5 contributing studies (Tufanaru et al., 2015). Funnel plots were generated to examine the extent of publication bias.

## Results

**Figure 1:**
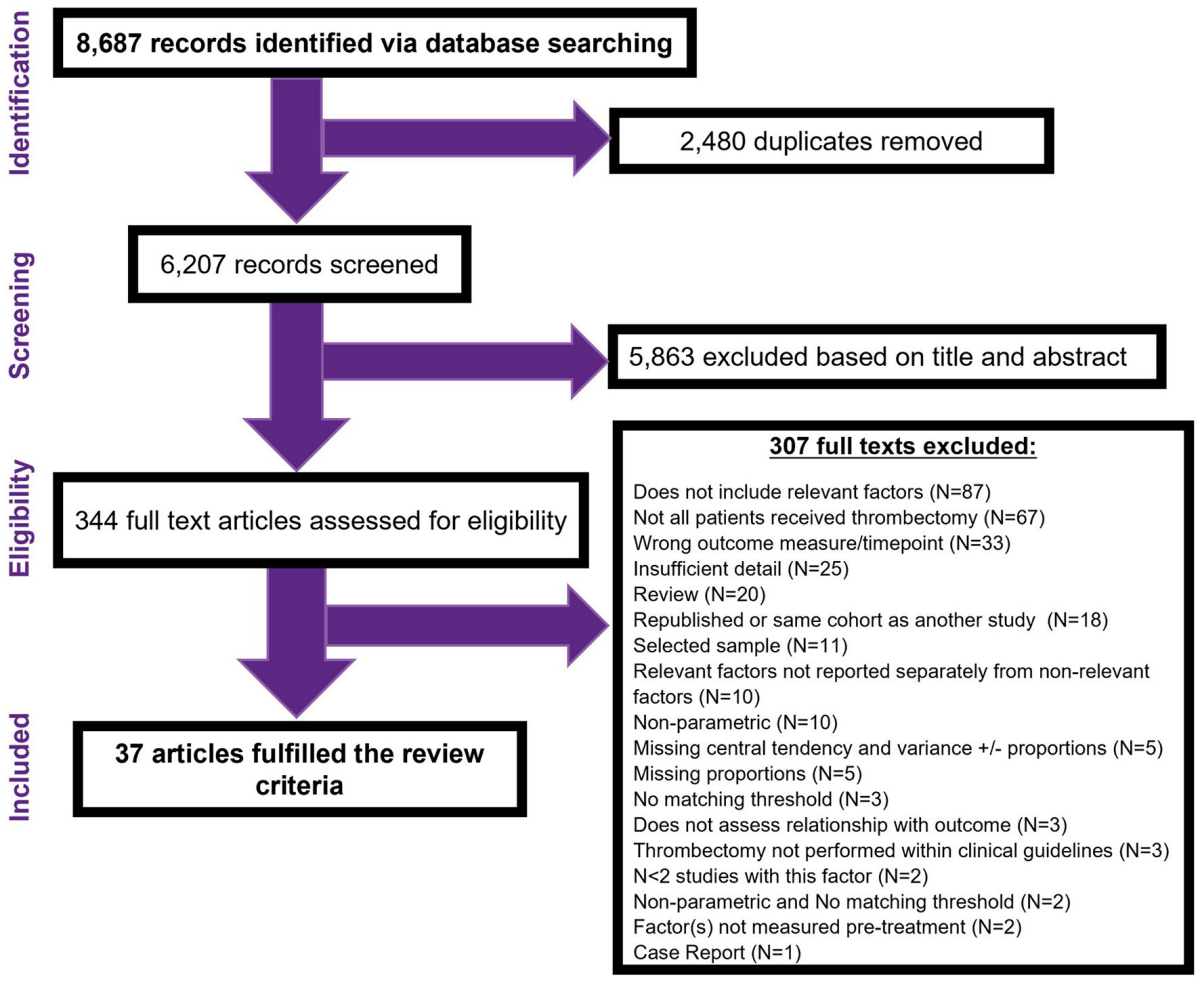
PRISMA flow diagram.

The search strategy identified 8,687 records. After removing 2,480 database duplicates, the title and abstract of 6,207 records were screened. This led to examination of 344 full text articles, 37 of which were eligible for inclusion (Figure 1).

Of the 307 excluded full-text articles, the primary reasons for ineligibility were as follows: Ninety-Nine (32%) studies did not include or individually report relevant factors/measurement timepoints. Not all patients received thrombectomy/thrombectomy within clinical guidelines in 70 (23%) studies. Forty-seven (15%) studies did not present sufficient detail to judge eligibility or include in the analysis. Thirty-three (11%) studies did not include our selected primary outcome measure/timepoint. Twenty (7%) studies were review articles. Eighteen (6%) studies were duplicate reports (e.g., conference abstracts of full texts articles or reports of the same factor(s) on the same cohort of patients). Eleven (4%) studies reported selected patient subgroups only. Five (2%) studies were excluded for having unique prognostic factors (*N* < 2). Three (1%) studies did not directly assess the relationship between the prognostic factor(s) and outcome measure. One (1%) paper was a single case study.

### Summary of included studies

Study summary information is detailed in Table 2.

**Table 2:** Summary of included studies.

**Author(s)**	**Country**	**Total number of patients**	**Study design**	**Inclusion criteria**	**Prognostic factor (s)**	**Vascular territory occluded**	**Thrombect-omy time window**	**Thrombect-omy technique**	**Bridging Thrombo-lysis (%)**	**Other Outcome measure(s) (Data type)**	**Other Outcome measure time point(s)**
**Primary Post-Thrombectomy Outcome Measure: Dichotomised Modified Rankin Score (mRS) - Favourable (mRS 0-2) and Unfavourable (mRS 3-6) 3-month Functional Outcome**											
Shriki et al. (2020)	United States	134	Cohort	Age >/ = 18; Transferred from another hospital and treated with thrombectomy; Available transportation records	BG	Mix	Not reported	Not reported	67.91%	Not Applicable	N/A
Ozdemir et al. (2015)	Turkey	70	Cohort	Age 18-80; Presentation < 6h onset for anterior and < 8 h for basilar LVO; NIHSS >/ = 10 at admission (for patients contraindicated to thrombolysis) or pre-thrombolysis with no dramatic post-thrombolysis clinical improvement (NIHSS reduced >/ = 8 points); No severe allergy to contrast medium or nitol; Pre-stroke independence (mRS < / = 2); No advanced terminal illness; No presumed septic embolus or suspicion of bacterial endocarditis; Clinical presentation of SAH even if negative on initial CT; Baseline glucose >50 mg/dL or < 400 mg/dL; Platelets >100000; No CT evidence of intraparenchymal tumour, intracranial haemorrhage or large (>1/3 MCA) regions of clear hypodensity at baseline; ASPECTS >5; No CT/MRI evidence of extensive brainstem lesions	BG	Mix	Standard	Stent-retriever	47.14%	Not Applicable	N/A
Suissa et al. (2020)	France	41	Cohort	MCA (M1) or tandem (MCA M1/ICA) LVO completely recanalised (mTICI 2a or 3) < / = 6 h symptom onset; NIHSS >6; ASPECTS >/ = 6; Absence of parenchymal haematoma	BG	Anterior Circulation	Standard	Unclear	51.20%	Not Applicable	N/A
Shi et al. (2014)	China	109	Cohort	Age >/ = 18; TREVO/TREVO2 age limit 85 and NIHSS 30/29 (note: MERCI omitted); Angiography confirmed anterior or posterior LVO; Ineligible or refractory to thrombolysis < 40.5 h; NIHSS >8; Thrombectomy < 8h symptom onset	BG	Mix	Extended	Stent-retriever	490.56%	Not Applicable	N/A
Broocks et al. (2020)	Germany	178	Cohort	Multimodal CT (NECT; CTA; CTP) confirmed distal ICA or MCA LVO patients < 6h from symptom onset; Visually evident early infarct lesion (ischaemic hypoattenuation on admission NECT or perfusion lesion with reduced CBV on CTP); Treated with thrombectomy with TICI score documented; Symptom onset-to-imaging time and NIHSS documented; Absence of ICH and pre-existing infarctions on admission NECT; No severe motion artefacts	BG	Anterior Circulation	Standard	Not reported	Not reported	Not Applicable	N/A
Zi et al. (2017)	China	698	Cohort	Age >/ = 18; CTA/MRA/DSA confirmed anterior LVO treated with thrombectomy; Absence of concomitant aneurysm; arteriovenous malformation or posterior occlusion; Complete critical baseline data available; 2014–2016	BG	Anterior Circulation	Extended	Mix	32.80%	Not Applicable	N/A
Lasek-Bal et al. (2022)	Poland	417	Cohort	LVO patients treated with thrombectomy < 6 h from onset with complete medical data from 2019 and 2021	BG	Mix	Standard	Primary Combined Approach (stent-retriever and aspiration-catheter) with balloon catheter	60.91%	Not Applicable	N/A
Karamchandani et al. (2022)	United States	57	Cohort	Acute BAO LVO evaluated with pre-treatment CTP and treated with thrombectomy 2017–2021	BG	Posterior Circulation	Extended	Primary Combined Approach (stent-retriever and aspiration-catheter)	28.07%	Not Applicable	N/A
Goyal et al. (2018)	United States	231	Cohort	CTA confirmed emergent LVO patients presenting < 6 h symptom onset and treated with thrombectomy; For 6-12h since symptom onset, advanced imaging was used (ASPECTS >/ = 6 and/or good collaterals on CTA)	BG	Mix	Extended	Mix	63.00%	Shift in ordinal mRS; Mortality and Survival (Binary)	3 months
Kim et al. (2018)	South Korea	309	Cohort and RCT	Angiography confirmed proximal LVO with moderate to severe neurological deficits treatable with thrombectomy < 8 h from symptom onset (plus SWIFT/SWIFT-PRIME/STAR criteria)	BG	Anterior Circulation	Standard	Mix	62.80%	Excellent (mRS 0-1) and Non-excellent (mRS 2–6) Functional Outcome (Binary); Ordinal mRS; Mortality and Survival (Binary)	3 months
Genceviciute et al. (2022)	Switzerland	1,020	Cohort	DSA confirmed Anterior LVO (ICA, Carotid terminus, M1/M2 MCA, or tandem ICA and M1/M2 occlusions) AIS treated with thrombectomy in accordance with clinical guidelines 2015–2020	BG	Anterior circulation	Extended	Stent-retriever	460.57%	Excellent (mRS 0–1) and Non-excellent (mRS 2-6) Functional Outcome (Binary); Ordinal mRS; Mortality and survival (Binary)	3 months
Huo et al. (2019)	China	149	Cohort	Age >18; acute anterior LVO treated with stent-retriever (Solitaire) thrombectomy < 12h symptom onset; Acute symptoms present >/ = 30 min with no significant improvement before treatment; Pre-stroke independence (mRS >/ = 1); NIHSS >/ = 8 and < 30; Absence of moderate/large ischaemic core on NCCT/DWI (extensive early ischaemic changes: ASPECTS < 7 or lesion volume >50 ml); 2015	BG	Anterior circulation	Extended	Stent-retriever	16.77%	Excellent (mRS 0–1) and Non-excellent (mRS 2–6) Functional Outcome (Binary); Mortality and Survival (Binary); Favourable (Barthel Index (BI) score 95–100) and Unfavourable (BI 0–95) Functional Outcome (Binary); Dramatic Neurological Improvement (DrNI: NIHSS reduction or NIHSS 0–2) and No DrNI (No NIHSS reduction or NIHSS >2) (Binary)	24 h (NIHSS) and 3 months (mRS, BI and Mortality)
Cao et al. (2021)	China	101	Cohort	Age >/ = 18; CTA/MRA confirmed BAO treated with thrombectomy < 24h symptom onset; Pre-stroke independence (mRS < / = 2); No surgery or trauma within previous 2 months; Absence of ICH; No history of ICH, SAH, tumour or arteriovenous malformation; Absence of large infarct core (>2/3 midbrain, pons or either cerebellar hemisphere); No vital organ dysfunction; Absence of bleeding tendency; No voluntary abandonment of treatment for iatrogenic purposes	BG	Posterior Circulation	Extended	Primary Combined Approach (stent-retriever and aspiration-catheter)	7.80%	Mortality and Survival (Binary)	3 months
Rinkel et al. (2020)	Netherlands	2,908	Cohort	Age >/ = 18; MR CLEAN registry 2014–2017; Admission glucose available; Anterior LVO	BG	Anterior circulation	Not reported	Mix	75.17%	Mortality and Survival (Binary)	3 months
Bouslama et al. (2018)	United States	931	Cohort	Anterior LVO patients treated with thrombectomy	BG	Anterior Circulation	Not reported	Mix	Not reported	Mortality and Survival (Binary)	3 months
Huo et al. (2016)	China	36	Cohort	Age >/ = 18; CTA/MRA confirmed acute BAO with atherosclerotic large artery, embolic or thrombogenic aetiology; Indicated for thrombectomy and treated with the Solitaire device 2012–2015; Absence of ICH on imaging; NECT or DWI confirmed favourable infarct core (early ischaemic changes in >2/3 of the area of the pons and midbrain); No allergy to contrast; No history of ICH, sICH, arteriovenous malformation or tumour; Absence of severe pre-stroke disability (mRS < / = 3); Absence of renal failure (creatinine < 2.0 mg/dL or glomerular filtration rate >30 mL/(minutesx1.73 m2)	BG	Posterior Circulation	Extended	Stent-retriever	36.11%	Mortality and Survival (Binary)	3 months
Nisar et al. (2021)	United States	188	Cohort	Anterior LVO patients treated with thrombectomy; Available 3-month outcome data; 2015–2020	BG	Anterior Circulation	Not reported	Not reported	53.70%	Mortality and Survival (Binary)	3 months
Chen et al. (2020)	China	248	Cohort	Age >/ = 18; CTA/MRA/DSA confirmed MCA or ICA LVO; Pre-stroke independence (mRS < / = 2); No severe renal disease, hepatic disease, cardiac insufficiency, tumour or autoimmune disease	BG; DBP; SBP	Anterior Circulation	Not reported	Stent-retriever	67.70%	Not Applicable	N/A
Wu et al. (2019)	China	118	Cohort	Anterior LVO treated with thrombectomy 2014–2018; Absence of malignant tumour(s), neurological diseases or previous stroke; Primary treatment at study hospital; Complete outcome data	BG; DBP; SBP	Anterior circulation	Not reported	Stent-retriever	Not reported	Not Applicable	N/A
Jiang et al. (2015)	United States	89	Cohort	CTA/MRA/DSA confirmed acute LVO treated with Solitaire thrombectomy (+/- thrombolysis and angioplasty) < 6 h symptom onset; NIHSS >/ = 10; Absence of hypodensity on CT/Multimodal MRI; When angiography unavailable, proximal occlusion defined as NIHSS >/ = 10, coma, hemiplegia, tetraparesis and aphasia; Absence of CT/MRI evidence of ICH or major infarct (acute ischaemic change >1/3 MCA or >100 ml tissue elsewhere); Absence of uncontrolled hypertension; Serious sensitivity to contrast agents	BG; DBP; SBP	Mix	Standard	Mix	28.09%	Mortality and Survival (Binary)	3 months
Sun et al. (2021)	China	212	Cohort	Acute ischaemic stroke patients undergoing thrombectomy	BG; DBP; SBP	Anterior Circulation	Not reported	Unclear	Not reported	Mortality and Survival (Binary)	3 months
Gordon et al. (2018)	United States	79	Cohort	Acute ischaemic stroke patients receiving thrombectomy using a retrieval stent; 2012–2016; No missing follow-up data	BG; SBP	Mix	Standard	Stent-retriever	Not reported	Not Applicable	N/A
Sallustio et al. (2019)	Italy	270	Cohort	CTA confirmed MCA +/- terminal ICA or proximal ICA combined with intracranial vessel; Groyne puncture < 6 h symptom onset; NIHSS >/ = 10; Not posterior circulation	BG; SBP	Anterior circulation	Standard	Unclear	550.56%	Not Applicable	N/A
Phuong et al. (2022)	Vietnam	49	Cohort	Acute MRA+DWI or CTA confirmed BAO LVO treated with thrombectomy within clinical guidelines 2018–2020	BG; Consciousness (GCS)	Posterior circulation	Extended	Primary combined approach (stent-retriever and aspiration-catheter) with balloon catheter	18.37%	Not Applicable	N/A
Yu et al. (2022)	China	304	Cohort	Age >/ = 18 years; CTA/DSA confirmed Anterior or Posterior LVO undergoing thrombectomy with successful reperfusion (mTICI 2b-3) from 2017 to 2021; No malignancy, autoimmune disease, severe renal insufficiency, liver disease, heart failure or other life-threatening comorbidities	DBP; SBP	Mix	Standard	Mix	300.56%	Not Applicable	N/A
Lin et al. (2022a)	China	312	Cohort	Age >/ = 18 years; Acute anterior ICA, MCA (M1/M2) LVO patients treated with stentretriever and/or aspiration catheter thrombectomy within 6 h or 6–24 h with perfusion mismatch (within clinical guidelines) (3) successful recanalization (mTICI 2b or 3); Prestroke independence (mRS 0-2); < / = 1 missing data point; 3-month outcome data available	DBP; SBP	Anterior Circulation	Extended	Primary Combined Approach (stent-retriever and aspiration-catheter)	44.23%	Not Applicable	N/A
Zhang et al. (2022)	China	258	Cohort	Age >/ = 18 years; DSA confirmed LVO (ICA, ACA, MCA, VBA) patients undergoing thrombectomy within 24 h of onset (6 h standard or longer with perfusion mismatch); Pre-stroke independent (mRS 0–1); Receiving antithrombotic drugs post-treatment; Absence of baseline or 24 h haemorrhage; Available baseline, laboratory, pre-/post-operative neuroimaging and 3-month outcome data	DBP; SBP	Mix	Extended	Not reported	50.00%	Not Applicable	N/A
Zeng et al. (2022)	China	110	Cohort	Age < / = 80; DSA confirmed unilateral ICA/MCA (M1 or M2) LVO AIS treated with thrombectomy < 6 h from onset (or up to 24 h with imaging mismatch) in accordance with clinical guidelines with successful recanalization (mTICI 2b-3) between 2016–2019; Pre-stroke independence (mRS 0–2); No	DBP; SBP	Anterior circulation	Extended	Not reported	51.82%	Not applicable	N/A
				history of intracranial haemorrhage, venous malformations, aneurysms, tumours or neurological comorbidities; No high risk of bleeding or haematological conditions; No severe vital organ failure; Life expectancy >1year							
Lin et al. (2022b)	China	84	Cohort	Age >/ = 18 years; BAO patients successfully recanalised (mTICI 2b-3) with thrombectomy from 2016–2021; Pre-stroke independent (mRS 0–2); Available baseline CT and outcome data	DBP; SBP	Posterior circulation	Extended	Not reported	38.10%	Not applicable	N/A
Li et al. (2022)	China	329	Cohort	Age >/ = 18 years; DSA/CTA confirmed Anterior LVO patients undergoing thrombectomy consecutive patients with from 2018–2019. NIHSS ≥3; Laboratory measurements available; No severe renal impairment (creatinine ≥ 180 mmol/L) or anaemia (haemoglobin < 100 g/L)	DBP; SBP	Anterior Circulation	Extended	Not reported	13.68%	Not Applicable	N/A
Anadani et al. (2019)	United States	1,245	Cohort	Anterior LVO successfully (mTICI >/ = 2b) treated with thrombectomy; Admission ASPECTS >6; Pre-stroke independence (mRS < / = 2)	DBP; SBP	Anterior Circulation	Not reported	Mix	65.86%	Mortality and Survival (Binary)	3 months
Cho et al. (2019)	South Korea	378	Cohort	Anterior LVO patients < 8 h symptom onset with 'small to moderate' infarct cores; Non-minor stroke (NIHSS >4); Absence of terminal medical conditions (e.g., malignancy or pre-stroke disability mRS >1)	DBP; SBP	Anterior Circulation	Extended	Unclear	58.20%	Mortality and Survival (Binary)	3 months
Goyal et al. (2017)	United States	116	Cohort	Anterior LVO patients treated with thrombectomy	DBP; SBP	Anterior Circulation	Not reported	Mix	64.66%	Mortality and Survival (Binary); Infarct Volume (Ratio)	In-Hospital (FIV and Mortality) and 3 months (Mortality)
Diprose et al. (2020)	New Zealand	432	Cohort	Thrombectomy patients	SBP	Mix	Not reported	Not reported	53.00%	Mortality and Survival (Binary)	3 months
Pinho et al. (2021)	Germany	489	Cohort	Age >/ = 18; Anterior LVO treated with thrombectomy 2012–2017; Available 3-month outcome data	AF	Anterior Circulation	Not reported	Mix	62.80%	Not Applicable	N/A
Soize et al. (2013)	France	59	Cohort	Acute ischaemic stroke patients treated with Solitaire FR thrombectomy 2010–2012	AF	Mix	Not reported	Stent-retriever	72.90%	Mortality and Survival (Binary)	3 months
Costalat et al. (2012)	France	50	Cohort	Clinical and MRI confirmed proximal LVO (MCA; ICA; BA); Thrombectomy < 6 onset for anterior and < 24 for posterior LVO; ASPECTS >5; No spontaneous NIHSS improvement after MRI; For POCS, no extensive brain stem lesions on DWI	Consciousness (GCS)	Mix	Standard	Primary combined approach (stent-retriever and aspiration-catheter) with balloon catheter	80.00%	Not applicable	N/A

ACA, Anterior Cerebral Artery; AF, Atrial Fibrillation; AIS, Acute Ischaemic Stroke; ASPECTS, Alberta Stroke Programme Early CT Score; BA, Basilar Artery; BAO, Basilar Artery Occlusion; BG, Blood Glucose; CBV, Cerebral Blood Volume; CT, Computed Tomography CTA, Computed Tomography Angiography; CTP, Computed Tomography Perfusion; DBP, Diastolic Blood Pressure; DSA, Digital Subtraction Angiography; DWI, Diffusion Weighted Imaging; GCS, Glasgow Coma Scale; ICA, Internal Carotid Artery; ICH, Intra Cerebral Haemorrhage; LVO, Large Vessel Occlusion; M1, MCA branch 1; M2, MCA branch 2; MCA, Middle Cerebral Artery; MERCI, Mechanical Embolus Removal in Cerebral Ischaemia; MRA, Magnetic Resonance Angiography; MRI, Magnetic Resonance Imaging; MR CLEA*N*, Multicentre Randomised Clinical Trial of Endovascular Treatment for Acute Ischaemic Stroke in the Netherlands; mTICI, modified Thrombolysis in Cerebral Infarction Scale; N/A, Not Applicable; NCCT, Non Contrast-Enhanced Computed Tomography; NECT, Non-contrast Enhanced Computed Tomography; NIHSS, National Institute of Health Stroke Scale; POCS, Posterior Circulation Stroke; SAH, Subarachnoid Haemorrhage; SBP, Systolic Blood Pressure; sICH, symptomatic Intracerebral Haemorrhage; STAR, Solitaire Flow Restoration Thrombectomy for Acute Revascularization; SWIFT, Solitaire With the Intention for Thrombectomy; VBA, Vertebral Basilar Artery.

### Basic study information

Of 37 included studies, the corresponding author of each was based in the following countries: China [*N* = 14 (38%)], United States [*N* = 9 (24%)], France [*N* = 3 (8%)], Germany [*N* = 2 (5%)], South Korea [*N* = 2 (5%)], Italy (*N* = 1), Netherlands (*N* = 1), New Zealand (*N* = 1), Poland (*N* = 1), Switzerland (*N* = 1), Turkey (*N* = 1) and Vietnam (*N* = 1). All studies were published after 2012, with the majority from 2019-2022 [*N* = 25 (68%)].

### Study design

All but one of the studies were retrospective cohort design [*N* = 36 (97%)] and the remaining study was a combined cohort and randomised controlled trial (RCT) [*N* = 1 (3%)]. Settings were described as Comprehensive Stroke Centres [*N* = 29 (81%)] or Tertiary Hospitals [*N* = 8 (19%)]. Blinding was not reported in 28 (76%) studies, and it was only stated that outcome assessors were blinded to baseline information in 7 (18%) or were not blinded in 2 (5%) studies. The average risk of bias was low overall [*N* = 34 (92%), mean 12.23/14] and was similar in reports of significant [*N* = 25 (43.1%), mean 12.68] and non-significant [*N* = 32 (55.2%), mean 11.86] findings (studies reported on multiple factors). The three (8%) remaining studies had a moderate risk of bias.

### Treatment context

Ten (27%) studies included patients treated within traditionally standard thrombectomy time windows (i.e. <6 h stroke onset for anterior, <24 h for posterior circulation stroke), whilst 14 (38%) included patients treated within the extended time window (i.e. 6–24 h for anterior circulation stroke with eligibility informed by advanced imaging) (National Institute for Health and Care Excellence, 2019; Sentinel Stroke National Audit Programme, 2023). The treatment time window was not reported in 13 (35%) studies. Twenty (54%) studies included patients with an occlusion in the anterior vascular territory only, five (14%) included posterior circulation occlusions only and 12 (32%) included both territories. The majority of studies reported either a mix of thrombectomy devices, usually individualised to the case [*N* = 10 (27%)], or included only the use of stent-retrievers [*N* = 9(24%)]. Three (8%) studies reported on the primary combined approach (aspiration-catheter and stent-retriever) with balloon catheter and three (8%) without balloon-catheter. Eight (22%) and four (11%) did not report or were unclear (respectively) about the use of thrombectomy devices. Bridging thrombolytic treatment was reported as available by 32 (86%) studies, however, use varied greatly [Median 51.5% (range 7.8%−80%)]. Five (14%) studies did not report the proportion of patients receiving thrombolysis.

### Prognostic factors

Within the included studies, there were 58 reports of the relationship between physiological observations and our primary outcome measure (Supplementary Table S5), reflecting the fact that studies often reported on multiple factors. Some studies also included both categorical and continuous analyses of the same factor. Although many papers examining pre-treatment atrial fibrillation (AF) were identified in the literature search, the majority either included it only as a previously recorded comorbidity or mixed patients with comorbid and acutely Electrocardiogram (ECG) detected AF. The following pre-treatment physiological observations were included: continuous (mmol/L) blood glucose (BG) (*N* = 19), categorical blood/serum glucose (hyperglycaemia >/ = 7.8mmol/L) (*N* = 6), continuous (mmHg) systolic blood pressure (SBP) (*N* = 16), continuous (mmHg) diastolic blood pressure (DBP) (*N* = 13), categorical ECG evidence of atrial fibrillation (AF) (*N* = 3) and continuous conscious level using the Glasgow Coma Scale (GCS) (*N* = 2). In 35 (95%) studies, the pre-thrombectomy prognostic factor measurement timepoint was described as “admission” and the remaining 2 (5%) as “pre-operative.”

### Outcome measures

Included studies were required to report favourable (0–2) vs. unfavourable (3–6) mRS at 3 months post-thrombectomy, but many also described multiple thrombectomy outcome measures at multiple timepoints. Other variations of mRS (i.e., ordinal mRS and different dichotomisations) and other non-mRS outcome measures were reported alongside our defined primary mRS outcome measure, leading to a total of 89 individual outcome results across factors. The frequency of all outcome measures is listed in Supplementary Table S5, with timepoints and results reported in Supplementary Tables S6-S10.

### Glucose

Of 24 studies (8,405 total patients) assessing the relationship between pre-treatment glucose and outcome, there were 19 reports (3,122 total patients) of glucose as a continuous variable (10 blood glucose, four serum glucose and five not specified) and six reports (5,481 total patients) of glucose as a categorical variable with a binary threshold of hyperglycaemia (>/ = 7.8 mmol/L) vs. no hyperglycaemia (<7.8 mmol/L) (four serum glucose, two not specified and one blood glucose). One study was included in both categorical and continuous analyses (Huo et al., 2019). Within the included continuous studies, three had ineligible categorical data due to missing proportions (*N* = 1), no matching threshold (*N* = 1) and no defined threshold (*N* = 1). Continuous data could not be included from three included categorical studies due to missing parametric data. The average number of patients per study was 350 [450 for 17 (70.83%) studies with significant findings and 107 for 7 (29.17%) non-significant studies]. The risk of bias (Modified QUIPS) was low overall (mean 11.96/14) regardless of whether results showed a significant difference (mean 12/14) or were non-significant (mean 11.86/14). Further details on glucose studies, as well as alternative outcomes, are reported in Supplementary Table S6.

### Continuous (ratio) glucose (mmol/L)

The funnel plot (Supplementary Figure S1) was fairly symmetrical; however, studies were significantly heterogeneous (*I*^2^ = 66%, Tau^2^ = 0.39, Chi^2^ = 53.55 (df = 18), *p* < 0.0001) and a random effects model was applied.

**Figure 2:**
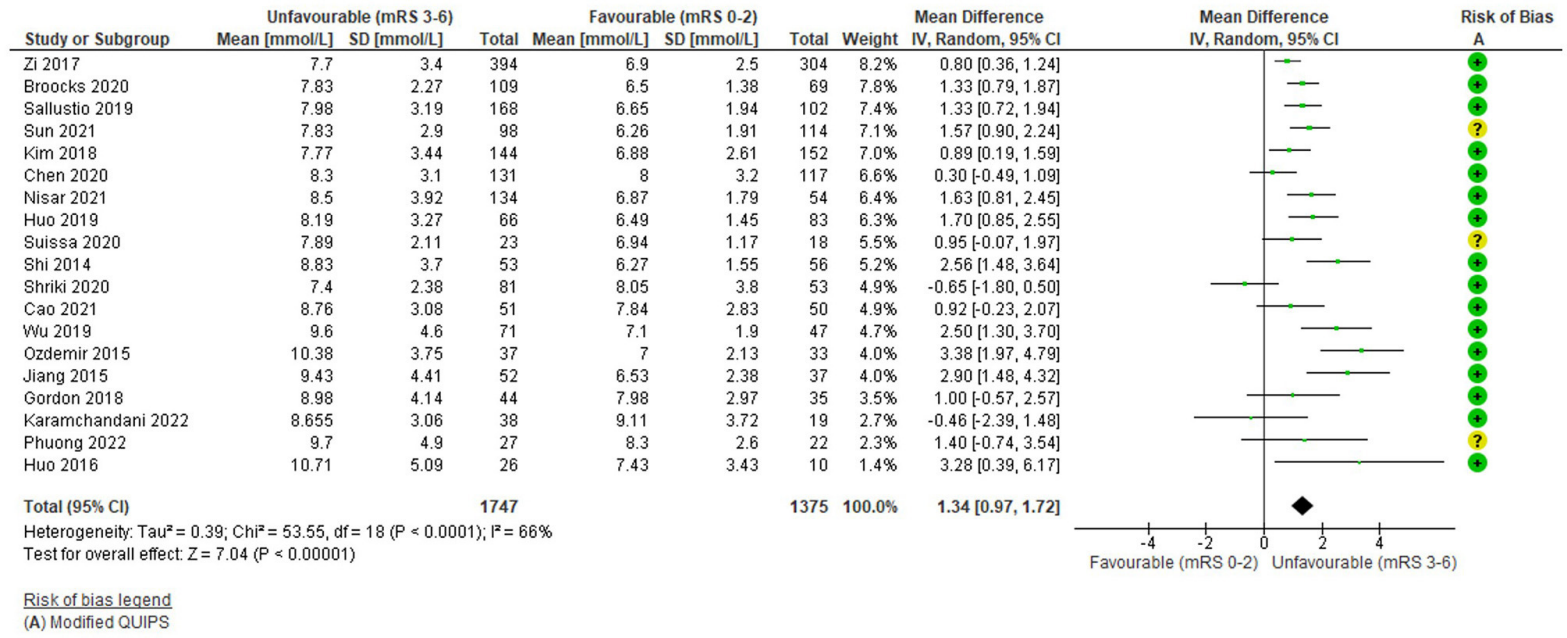
Effect of pre-treatment glucose (mmol/L; continuous) on outcome (mRS).

Overall, higher pre-thrombectomy blood/serum glucose was significantly associated with an unfavourable functional outcome: WMD = 1.34 mmol/L (95%CI 0.97 to 1.72; Z = 12.3) *p* < 0.00001 (Figure 2). Most [*N* = 13/19 (68%)] individual studies reported significantly higher glucose in unfavourable outcome groups (Shi et al., 2014; Jiang et al., 2015; Ozdemir et al., 2015; Zi et al., 2017; Kim et al., 2018; Huo et al., 2019; Sallustio et al., 2019; Wu et al., 2019; Broocks et al., 2020; Chen et al., 2020; Suissa et al., 2020; Nisar et al., 2021; Sun et al., 2021) (Supplementary Table S6), including the highest weighted study [(Zi et al., 2017); *N* = 698], although the WMD was not large. This was the case for most high weighted studies, apart from Chen et al. (2020). Shriki et al. (2020) and Karamchandani et al. (2022) displayed non-significant differences in the opposite direction but were small, low weighted studies. Risk of bias was low overall (mean 11.94/14) and studies with a moderate risk of bias were evenly distributed in terms of their weights. Other outcomes were generally consistent with the overall effect, with significantly greater mortality, higher ordinal mRS and fewer favourable outcomes (Supplementary Table S6).

### Categorical (Binary) Glucose (Hyperglycaemia >/ = 7.8mmol/L vs. No Hyperglycaemia <7.8 mmol/L)

The funnel plot (Supplementary Figure S2) was fairly symmetrical; however, studies were significantly heterogeneous (*I*^2^ = 58%, Tau^2^ = 0.05, Chi^2^ = 11.81 (df = 5), *p* = 0.04) and a random effects model was applied.

**Figure 3:**
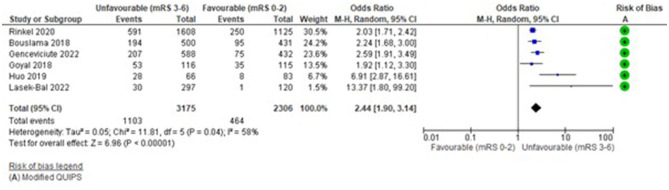
Effect of pre-treatment hyperglycaemia (Glucose >/ = 7.8 mmol/L) vs. no hyperglycaemia (<7.8 mmol/L) on outcome (mRS).

Figure 3 demonstrates that hyperglycaemia >/ = 7.8mmool/L had a significant association with unfavourable functional outcome: OR = 2.44 (95%CI 1.9 to 3.14, Z = 6.96), *p* < 0.00001. This was consistent for all six studies (Bouslama et al., 2018; Goyal et al., 2018; Huo et al., 2019; Rinkel et al., 2020; Genceviciute et al., 2022; Lasek-Bal et al., 2022) (Supplementary Table S6). The highest weighted studies (Bouslama et al., 2018; Rinkel et al., 2020; Genceviciute et al., 2022) showed a clear association and lower weighted studies had greater mean differences (Huo et al., 2019; Lasek-Bal et al., 2022). Risk of bias was low (mean 12.08/14). Other outcomes reported by these studies were consistent with this direction of effect, with significantly greater mortality, higher ordinal mRS and fewer favourable (mRS 0–2) functional outcomes (Supplementary Table S6).

### Systolic blood pressure

Sixteen studies (4,400 total patients) assessed the relationship between pre-treatment SBP (mmHg) as a continuous variable and 3-month functional outcome. The average number of patients per study was 276 (209 for 5 studies with significant findings and 293 for 10 studies with non-significant findings). The risk of bias (Modified QUIPS) for the SBP studies was low overall (mean 12.5/14), and similar for studies with significant (mean 12.4/14) and non-significant (mean 12.6/14) differences in functional outcome. One study had a moderate risk of bias (Sun et al., 2021). Further details on SBP studies, as well as alternative outcomes, are reported in Supplementary Table S7.

The funnel plot (Supplementary Figure S3) was fairly symmetrical; however, studies were significantly heterogeneous [I^2^ = 43%, Tau^2^ = 7.44, Chi^2^ = 26.45 (df = 15), *p* = 0.03] and a random effects model was applied.

**Figure 4:**
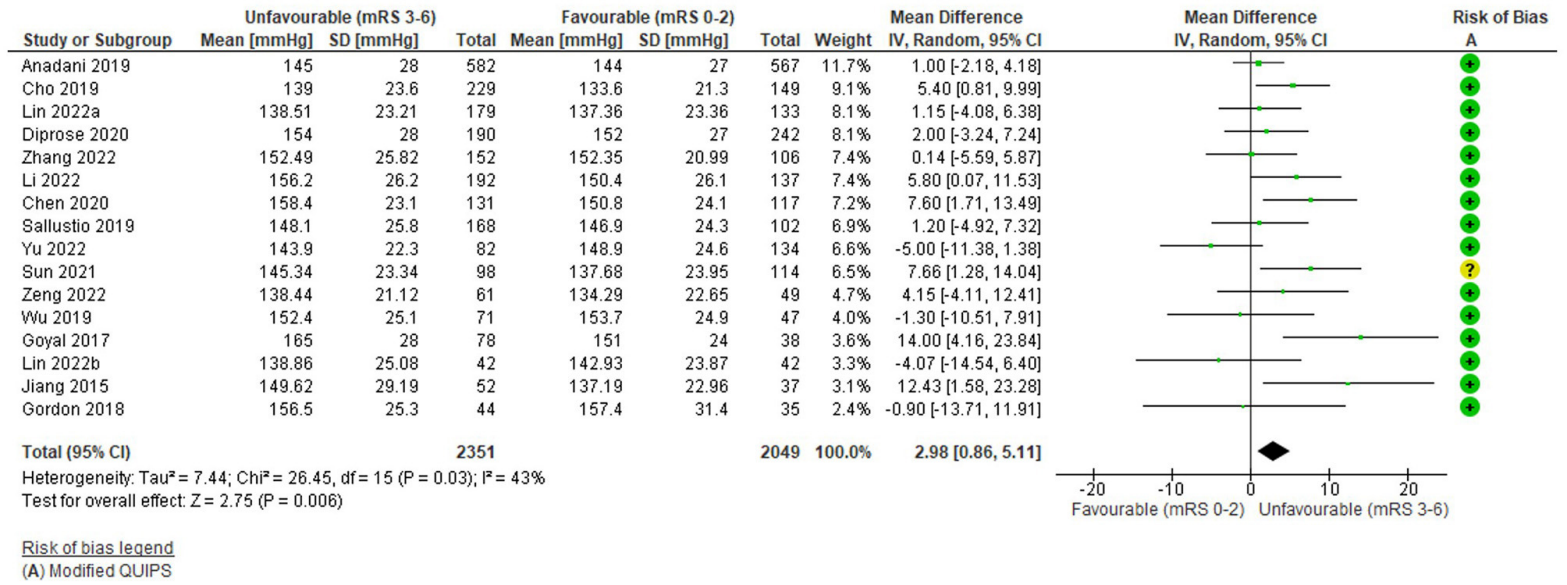
Effect of pre-treatment SBP (mmHg; continuous) on outcome (mRS).

Figure 4 demonstrates that higher admission SBP had a small, significant association with unfavourable functional outcome: WMD = 2.98 mmHg (95%CI 0.86 to 5.11, Z = 2.75), *p* = 0.006. Reflected in the forest plot, four highly weighted studies and one low weighted study demonstrated a small but significant difference in outcome (Jiang et al., 2015; Goyal et al., 2017; Cho et al., 2019; Chen et al., 2020; Sun et al., 2021) (Supplementary Table S7). However, this small effect was not evenly distributed across studies and the highest weighted studies (Anadani et al., 2019; Diprose et al., 2020; Lin et al., 2022a), reported non-significant associations (Supplementary Table S7). Four studies demonstrated non-significant effects in the opposite direction (Gordon et al., 2018; Wu et al., 2019; Lin et al., 2022b; Yu et al., 2022; Zhang et al., 2022). However, the overall direction of the effect was consistent with other outcomes reported by these studies, including mortality and final infarct volume (Supplementary Table S7).

### Diastolic blood pressure

Thirteen studies (3,614 total patients) assessed the relationship between pre-treatment DBP (mmHg) as a continuous variable and 3-month functional outcome (mRS 0–2 vs. 3–6). The average number of patients per study was 271 (169 for 2 studies with significant findings and 290 for 11 studies with non-significant findings). The risk of bias (Modified QUIPS) for the DBP studies was low overall (mean 12.6/14) and similar for studies with significant (mean 12.8/14) and non-significant (mean 12.5/14) differences; only one study had a moderate risk of bias (Sun et al., 2021). Further detail on DBP studies, as well as alternative outcomes, is reported in Supplementary Table S8.

The funnel plot (Supplementary Figure S4) was fairly symmetrical and there was no significant heterogeneity (Tau^2^ = 0.04, *i*^2^ = 9%, Chi^2^ = 13.26 (df = 12), *p* = 0.35) but as heterogeneity was anticipated and study results were bidirectional, a random effects model was still applied.

**Figure 5:**
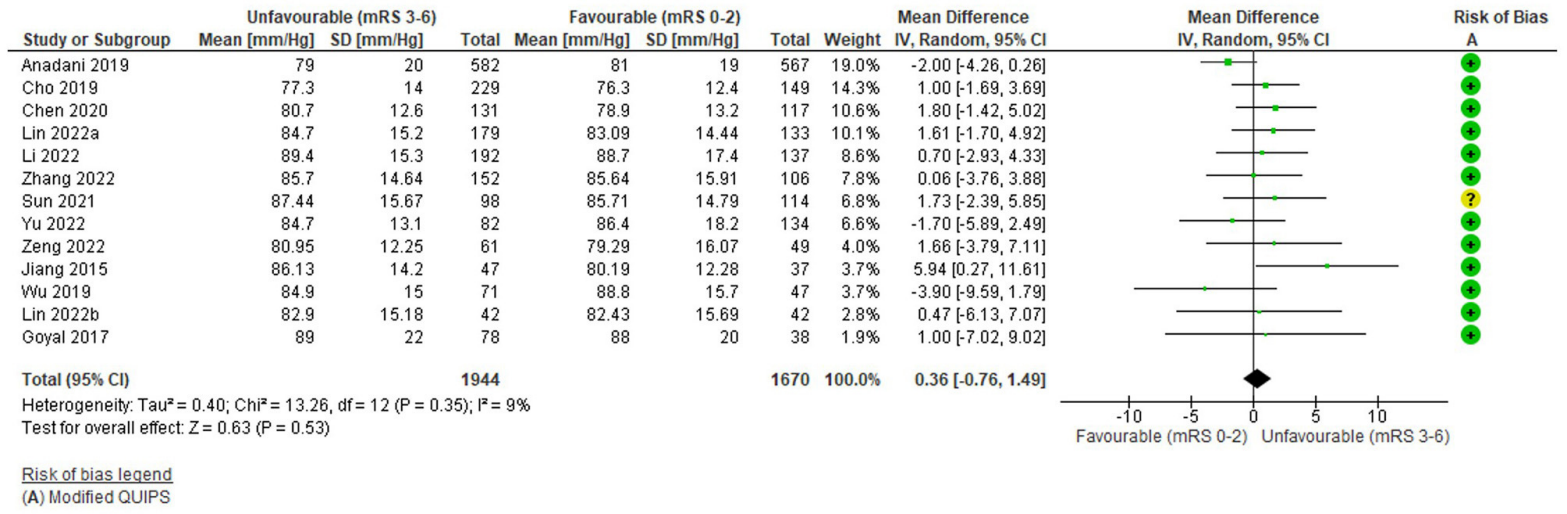
Effect of pre-treatment diastolic blood pressure (mmHg; continuous) on outcome (mRS).

Overall, there was no significant association between pre-treatment DBP and unfavourable outcome: WMD = 0.36 mmHg (95%CI −0.76 to 1.49, Z = −0.63), p = 0.53 (Figure 5). No study reported a significant difference in DBP between outcome groups (Supplementary Table S8) and this is reflected in the forest plot. Most studies demonstrated higher DBP in unfavourable outcome groups; however, three studies reported effects in the opposite direction (Anadani et al., 2019; Wu et al., 2019; Yu et al., 2022), two of which had also reported this for SBP (Wu et al., 2019; Yu et al., 2022). The study with largest mean difference, in favour of higher DBP in the unfavourable outcome group, had a low weight due to its small sample (Jiang et al., 2015; *N* = 84), whereas the highest weighted study was in the opposite direction (Anadani et al., 2019; *N* = 1149). Variation in the direction of effect was evenly distributed in terms of study weights. Findings were consistent with those reported for other outcomes, including no significant group differences for mortality or final infarct volume (Supplementary Table S8).

### Electrocardiogram (ECG) detected atrial fibrillation

Three studies (736 total patients) considered the relationship between AF detected on a pre-treatment ECG (present vs. absent) and 3-month functional outcome (mRS 0-2 vs 3-6). The average number of patients per study was 245 (489 for one study with significant findings and 124 for 2 studies with non-significant findings). The risk of bias (Modified QUIPS) for AF was low for all studies (mean 12.7/14) and similar for studies with both significant (mean 13.5/14) and non-significant (mean 12.3/14) differences. Further detail on AF studies, as well as alternative outcomes, is reported in Supplementary Table S9.

The funnel plot (Supplementary Figure S5) was fairly symmetrical, although potentially biassed by few studies, there was no significant heterogeneity (Tau^2^ = 0, *I*^2^ = 0%, Chi^2^ = 1.38 (df = 2), *p* = 0.5) and there were fewer than five studies so a fixed effects model was applied.

**Figure 6:**
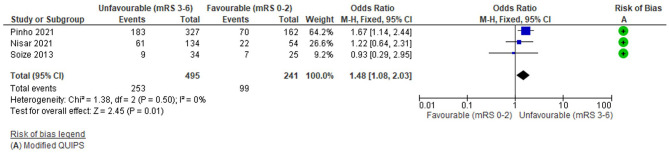
Effect of Pre-treatment ECG detected Atrial Fibrillation (binary) on outcome.

Overall, there was a significant association between pre-treatment AF and unfavourable functional outcome: OR = 1.48 (95%CI 1.08 to 2.03, Z = 2.45), *p* = 0.01 (Figure 6). The analysis was dominated by one study with 489 patients (Pinho et al., 2021) which reported a significant difference in outcome (Supplementary Table S9); the remaining studies were non-significant and this is reflected in the forest plot. One study reported a non-significant effect in the opposite direction (Soize et al., 2013) but this was weighted substantially lower (*N* = 59). Other outcomes reported by these studies reflected their small size, with no significant group differences for mortality (Supplementary Table S9).

### Level of consciousness

Two studies (99 total patients) assessed the relationship between pre-treatment conscious level, using Glasgow Coma Scale (GCS), and 3-month functional outcome (mRS 0-2 vs. 3-6). The risk of bias for the GCS studies was low overall (mean 10.8/14) but one (Phuong et al., 2022) had a moderate risk. Further details are reported in Supplementary Table S10.

The funnel plot (Supplementary Figure S6) was symmetrical, although the lack of studies suggests possible bias. There was no significant heterogeneity (Tau^2^ = 0, *I*^2^ = 0%, Chi^2^ = 0.04 (df = 1), *p* = 0.53) and there were *N* < 5 studies so a fixed effects model was applied.

**Figure 7:**
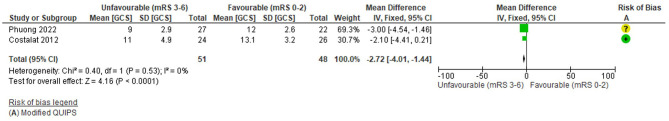
Effect of Pre-treatment Level of Consciousness (GCS; continuous) on outcome. ^*^Note that the x-axis is reversed to accommodate the direction of the GCS.

Overall, there was a significant association between lower pre-treatment conscious level recorded by GCS (lower score = lower conscious level) and unfavourable outcome: WMD = −2.72 points (95%CI −4.01 to −1.44, Z = 4.16), *p* < 0.0001 (Figure 7). Confidence intervals did not intersect 0 on the forest plot but studies had similarly small sample sizes and neither reported significant differences in outcome (Supplementary Table S10). The highest weighted study (Phuong et al., 2022) had a moderate risk of bias. There were no other outcome measures for the GCS studies (Supplementary Table S10).

## Discussion

This meta-analysis has demonstrated significant associations between basic clinical characteristics routinely recorded pre-thrombectomy and unfavourable outcome including higher blood/serum glucose, higher SBP, presence of AF and lower conscious level. It is important to recognise that the findings do not imply causal effects or that modification of the characteristics could change treatment effectiveness or prognosis but that their relationship with outcome could be useful for patient selection to avoid futile transfers and treatments.

Although our review only includes patients treated with thrombectomy, there have been previous reports in many different settings describing associations between baseline physiological characteristics and short or long term outcomes following acute stroke, which has led to their inclusion in prognostic models and scores (Saposnik et al., 2011; Fahey et al., 2018; Alaka et al., 2020). Although it might be expected that these characteristics are also relevant to the thrombectomy population, it is equally important to specifically consider whether their impact is the same as for the wider acute stroke population because relationships might be changed by selection for treatment (e.g., higher symptom burden) and/or the effects of thrombectomy plus associated interventions (e.g., blood pressure lowering post-treatment).

Prognostic scores have previously been constructed to predict outcomes after thrombectomy using different combinations of clinical information. A comprehensive overview and external validation of scores (Kremers et al., 2021) has shown that some have a good combination of discrimination and calibration for prediction of functional outcome for patients receiving thrombectomy treatment within 6.5 h of onset. For example, when applied retrospectively to a validation cohort of 3,156 thrombectomy registry patients including 1,193 (40.5%) with a 3-month mRS score of 0–2, the THRIVE-c and MR PREDICTS scores predicted outcome with an area under the curve of 0.74 (95%CI 0.72 to 0.76) and 0.80 (95%CI 0.78 to 0.81) respectively. However, these scores also incorporate additional clinical assessments which are not standard for generalists performing initial patient review (e.g., National Institutes of Health Stroke Scale) and/or detailed radiological information, so could not be used for very early triage. Concerns have also been raised about clinician reliance upon predictive scores for making high impact and complex treatment decisions such as thrombectomy, particularly because these cannot reflect all possible combinations of clinical information and they have usually been developed from specific patient populations with uncertain wider generalisability (Pan et al., 2018; Hamann et al., 2021; Kremers et al., 2021). Therefore, it is still valuable to understand the strength and direction of associations between individual clinical characteristics and outcomes, which are reflective of data pooled from similar studies across multiple real-world settings.

The review factors with most data were glucose and SBP. Elevated glucose had the strongest relationship with unfavourable outcomes, with similar results previously described following meta-analysis of data from key RCTs evaluating thrombectomy (Chamorro et al., 2019). The mechanism of this association is not yet clearly established and may be due to biochemical injury of the vascular endothelium and/or arterial smooth muscle resulting in reduced blood flow despite reperfusion, or a neurotoxic effect increasing infarct volume when restoration of oxygenated blood flow boosts the formation of free radicals because of the greater availability of glucose (Martini and Ta, 2007; Natarajan et al., 2011). However, intensive lowering of elevated blood glucose during acute ischaemic stroke does not appear to improve outcome (Johnston et al., 2019) and more investigation is required to determine whether glucose is a modifiable risk factor for unfavourable outcome in the thrombectomy population.

It is perhaps surprising that blood pressure did not have a stronger relationship with post-thrombectomy outcome when it has consistently been shown to be a prognostic factor during acute stroke and higher levels are associated with an increased risk of haemorrhage after reperfusion (Willmot and Leonardi-Bee, 2004; Appleton et al., 2016). There have also been reports of a potential U-shaped association between low and high SBP and unfavourable outcome (Leonardi-Bee et al., 2002). However, patients undergoing thrombectomy - and hence included in this review—have already had a degree of clinical screening to exclude individuals who may not benefit from treatment, including those with severe hypertension (Mathews, 2021). Other explanations for disagreement between studies in our analysis include the timing of reported measures (e.g., on admission or pre-thrombectomy when sedation may have been administered) and the effect of additional treatments. For instance, it is unknown whether some readings were performed after intravenous blood pressure lowering treatment or bridging thrombolysis which could affect pre-treatment blood pressure measures or enhance reperfusion and confound the association with post-thrombectomy outcome. Evidence on potentially adverse effects of blood pressure lowering suggest that it may only be useful for patient selection rather than as a modifiable factor (Yang et al., 2022). Indeed, a recent trial has shown that intensive lowering post-thrombectomy is associated with a poorer outcome (Nam et al., 2023). There are ongoing trials of blood pressure modification before and during thrombectomy which could be included in a future meta-analysis (Bath and Havard, 2021; Li and Zhao, 2022), including in the pre-hospital setting (Song, 2020).

Atrial fibrillation is a well-recognised marker associated with a poorer prognosis, an increased rate of medical complications and a higher in-hospital mortality (Steger et al., 2004). Within the thrombectomy treated population, patients with AF experienced worse outcomes despite similar rates of reperfusion, which is likely to be attributed to their greater age and greater number of comorbidities (Kobeissi et al., 2023). However, it has also been speculated that occlusions associated with AF are technically more difficult to remove during intra-arterial treatment (Staessens et al., 2021).

In line with expectations, there was an association between lower conscious level and unfavourable outcome. Most studies did not report this feature separately from the NIHSS, which was judged to be a specialist assessment, and so were not included. The GCS is widely used during initial emergency review, but as the speech and movement components can also be affected by stroke, it is likely to reflect symptom severity as well as consciousness (Weir and Bradford, 2003). There is a recent report of NIHSS being used reliably by trained ambulance personnel (Larsen et al., 2022), which may provide more valuable information for early triage of suspected stroke; however this may be difficult to sustainably implement into general paramedic practice in other settings.

It is important to acknowledge limitations in the review process and included studies. Nearly all data were obtained from registries and convenience cohorts, including some that pre-dated landmark RCTs which led to formal regulatory approval of thrombectomy (Goyal et al., 2016). However, despite little attempt at blinding and obvious heterogeneity causing concerns about generalisability, most had a low risk of bias and it is important that the data reflect real-world practice rather than clinical trials with strict inclusion criteria in high performing centres. Although an mRS dichotomisation of 0–2 vs. 3–6 is the most common approach for reporting thrombectomy treatment effects in clinical studies and enabled pooled analysis, it is a binary outcome and so does not allow examination of more complex associations e.g., it has been reported that there is a J shaped association between glucose and outcome (Rinkel et al., 2020; Genceviciute et al., 2022), with more favourable outcomes between 3.7 and 7.3 mmol/L (Ntaios et al., 2010). Also, it does not necessarily reflect treatment effectiveness if patients already had a high level of baseline dependency (mRS 3–6), although most studies only included patients who were independent pre-stroke (mRS 0–2). Robust analyses could not be undertaken for AF and GCS due to insufficient eligible studies and no studies reported any combinations of predictive factors to allow further explanation of relationships between them, e.g., SBP in the group with AF. Studies did not consistently report some variables which could also relate to outcome, such as the thrombolytic agent used and the time of administration and it was not possible to perform any sensitivity analyses based on these. It is also important to note that some studies did not separately report data for anterior and posterior circulation stroke and we took the decision to pool data across all vascular territories for the meta-analysis. Finally, as there are many other dominant factors which should be considered during thrombectomy treatment decisions, the factors described in this review should not be used in isolation to triage suspected stroke patients as they only had small effects on outcome.

The basic clinical factors included in our analysis are usually available during initial non-specialist assessment, and possibly could be used to refine selection of patients suitable for thrombectomy, such as during ambulance triage towards Comprehensive Stroke Centres. Reports of symptom-based pre-hospital redirection pathways confirm that not all patients with LVO arriving at CSC receive thrombectomy, and it is possible that some of these had unfavourable non-stroke characteristics (e.g., high systolic blood pressure) which might especially deter interventionists from treating patients with uncertain benefit (e.g., later in the time window and/or with a larger ischaemic core). For instance, in the Direct Transfer to Endovascular Center of Acute Stroke Patients with Suspected Large Vessel Occlusion in the Catalan Territory (RACECAT) trial (Pérez de la Ossa et al., 2022), amongst 482 intervention patients who were taken directly to a thrombectomy capable centre, 333 had recent onset LVO confirmed but only 235 (70%) received treatment, and it is possible that further consideration of additional prognostic information before transportation could reduce unnecessary patient displacement during implementation of this pathway model. There is little data so far which directly addresses this question, and only one study included in our meta-analysis reported data from the prehospital setting (Shriki et al., 2020). Further evaluation is needed of emergency care pathways which collect and use these characteristics. In England, the Specialist Pre-hospital Redirection for Ischaemic Stroke Thrombectomy (SPEEDY) trial (Shaw, 2022) is currently examining the clinical and cost-effectiveness of pre-hospital patient redirection to thrombectomy providers using a two-stage pre-hospital triage process during which ambulance personnel collect and communicate key information to specialist centres for a direct admission decision. The standard information checklist includes pre-hospital heart rate, blood pressure, oxygen saturations, blood glucose, temperature and conscious level, so the trial is expected to provide additional evidence about the value of these early basic measures for predicting thrombectomy outcome when LVO is present.

Future work in pre-hospital and hospital settings should also consider combining physiological factors with other non-stroke characteristics which may have a bearing on thrombectomy outcome such as frailty, pre-stroke dependency and comorbidities (Adamou et al., 2022; Tan et al., 2022; Barow et al., 2023). Although some have been included in previously published thrombectomy outcome scores (Kremers et al., 2021), especially pre-stroke dependency, it has not yet been demonstrated that they are accurate when used by non-specialist practitioners making early triage decisions towards thrombectomy providers. As it is likely that some factors will interact (e.g., patients with diabetes as a co-morbidity are likely to have high blood glucose), relationships between them may be complex and could also vary according to symptom severity and time since onset. Machine learning models are useful in this scenario and could help to create more accurate prognostic tools which are then embedded in software on portable devices to assist non-specialists when making early triage decisions and interventionists deciding whether to offer thrombectomy to patients with complex medical presentations (Thomas et al., 2021; Lin et al., 2022a). In future such models could potentially incorporate novel biomarker tests and/or technologies which are challenging for clinicians to interpret for individual patients, such as surface EEG (Montellano et al., 2021; Sutcliffe et al., 2022). The outputs would also facilitate discussion with patients and their families about the probability of a good outcome according to individual health and physiological profiles.

Finally, it is important that interventionists and stroke services undertake regular audit of their practice, including descriptions of baseline factors which may influence outcomes. As associations have been demonstrated between basic physiological characteristics and post-thrombectomy dependency, details of these variables should be included in audit reports and registries to illustrate any population variations between settings. Adjustment for the effects of these characteristics could also help to understand differences in outcomes between centres and so facilitate comparisons of care delivery in real world settings (Nie et al., 2023; Quandt et al., 2023).

## Conclusion

Basic physiological observations available during assessment by generalist emergency practitioners are associated with outcome after thrombectomy and might assist with early triage decisions, however these should not yet be considered as independent outcome predictors and associations do not imply that modification of factor(s) can change outcome. There were low numbers of cases for some factors and thrombectomy providers should continue to share data for pooled analysis and assist with evaluation of emergency care pathways which include basic characteristics during early selection of patients for treatment.

## Data availability statement

The original contributions presented in the study are included in the article/Supplementary material, further inquiries can be directed to the corresponding author.

## Author contributions

HL: Data curation, Formal analysis, Investigation, Methodology, Resources, Visualisation, Writing—original draft, Writing—review and editing. LS: Conceptualisation, Funding acquisition, Methodology, Supervision, Writing—review and editing. JM: Data curation, Writing—review and editing. AA: Data curation, Formal analysis, Writing—review and editing. PW: Writing—review and editing. GF: Writing—review and editing. MJ: Writing—review and editing. CP: Conceptualisation, Formal analysis, Funding acquisition, Methodology, Resources, Software, Supervision, Writing—original draft, Writing—review and editing.
