# Supplementary material for: Associations between basic physiological observations recorded pre-thrombectomy and functional outcome: a systematic review and meta-analysis
Source: Front Stroke. 2023 Oct 19;2:1283312. doi: 10.3389/fstro.2023.1283312 (PMC12802731; doi:10.3389/fstro.2023.1283312)
Supplement: Supplementary file 1 [file Data_Sheet_1.docx]

Supplementary Materials

Table S1. Search Strategy

**All databases:** Omit research before first intra-arterial device approved (1/8/4); limit to English Language only (abstracts); no protocols.

| **Medline (**Key Word and MeSH term combination): | |
| --- | --- |
| (SELECT MEDLINE DATABASES, NOT “IN PROGRESS”) | |
| Outcome | 1. (outcome* or disease course or followup or follow-up).mp. or Follow-Up Studies/ or exp Treatment outcome/ or medical futility/ or futil*.mp. or treatment failure/ or recover*.mp. or progress*.mp. or Glasgow coma scale/ or GCS.mp. or Glasgow outcome scale/ or GOS.mp. or (modified adj2 rankin).mp. or mRS.mp. or NIHSS.mp. or (Barthel adj2 (scale or index)).mp. or Cognition/ or cognit*.mp. or (Montreal adj2 (cognitive or assessment)).mp. or moca.mp. or "Quality of Life"/ or (quality adj2 life).mp. or disab*.mp. or dependen*.mp. or function*.mp. or Mortality/ or mortality.mp. or Death/ or death.mp. or surviv*.mp. or exp postoperative complications/ or complication*.mp |
| Timing and Setting | 1. (pre-thrombectomy or prethrombectomy or prestroke or pre-stroke or preexisting or pre-existing or premorbid* or pre-morbid* or preintervention or pre-intervention or pre-operative or preoperative or preprocedural or pre-procedural or ((before or prior) adj3 (stroke* or thrombectom* or hospital*)) or pre-hospital or prehospital or admission or preadmission or pre-admission or (emergency adj3 assess*) or (early adj3 assess*) or (acute adj3 assess*) or triag*).mp. or exp Comorbidity/ or ep.fs. |
| Population | 1. (Cerebrovascular accident or CVA or stroke).mp. or exp Stroke/ or ((brain or cerebral or cerebrovascular) adj3 (infarct* or isch?emia)).mp. 2. (Large vessel occlusion or LVO or large artery occlusion or LAO).mp. or exp Thrombectomy/ or Thrombectom*.mp. or (clot adj3 removal).mp. or (clot adj3 retriev*).mp. or (stent adj3 retriever).mp. or stentretriever.mp. or Embolectomy/ or embolectomy.mp. or ((surgical or mechanical) adj2 (reperfusion or thrombolysis or revasculari* or recanali*)).mp. or MT.mp. or Endovascular Procedures/ or endovascular.mp. or endo-vascular or EVT.mp. or (reperfusion adj2 (therapy or treatment)).mp. or (exp stroke/su and (reperfusion or revasculari* or recanali*).mp.) |
| Prediction | 1. Prognosis/ or Forecasting/ or (predict* or prognos* or forecast*).mp or (associat* adj4 (sever* or risk* or outcome* or surviv* or mortality)).mp or determinan*.mp. or exp Comorbidity/ or exp "Severity of Illness Index"/ or (likel* adj5 (sever* or outcome* or surviv* or mortality or associat*)).mp. or biomarker*.mp. |
| Combined Sets | 1. 1 AND 2 AND 3 AND 4 AND 5 |
| **EMBASE (via OVID) (**Key Word and MeSH term combination): | |
| Outcome | 1.       (outcome* or disease course or followup or follow-up).mp. or Follow-Up Studies/ or exp Treatment outcome/ or medical futility/ or futil*.mp. or treatment failure/ or recover*.mp. or progress*.mp. or Glasgow coma scale/ or GCS.mp. or Glasgow outcome scale/ or GOS.mp. or (modified adj2 rankin).mp. or mRS.mp. or NIHSS.mp. or (Barthel adj2 (scale or index)).mp. or Cognition/ or cognit*.mp. or (Montreal adj2 (cognitive or assessment)).mp. or moca.mp. or "Quality of Life"/ or (quality adj2 life).mp. or disab*.mp. or dependen*.mp. or function*.mp. or Mortality/ or mortality.mp. or Death/ or death.mp. or surviv*.mp. or exp postoperative complications/ or complication*.mp |
| Timing and Setting | 2.       (pre-thrombectomy or prethrombectomy or prestroke or pre-stroke or preexisting or pre-existing or premorbid* or pre-morbid* or preintervention or pre-intervention or pre-operative or preoperative or preprocedural or pre-procedural or ((before or prior) adj3 (stroke* or thrombectom* or hospital*)) or pre-hospital or prehospital or admission or preadmission or pre-admission or (emergency adj3 assess*) or (early adj3 assess*) or (acute adj3 assess*) or triag*).mp. or exp Comorbidity/ or ep.fs. |
| Population | 1. (Cerebrovascular accident or CVA or stroke).mp. or exp Stroke/ or brain ischemia/ or brain infarction/ or ischemic stroke/ or cerebral infarction/ or ((brain or cerebral or cerebrovascular) adj3 (infarct* or isch?emia)).mp. 2. (Large vessel occlusion or LVO or large artery occlusion or LAO).mp. or exp Thrombectomy/ or Thrombectom*.mp. or (clot adj3 (removal or retriev*)).mp. or (stent adj3 retriever).mp. or stentretriever.mp. or Embolectomy/ or embolectomy.mp. or ((surgical or mechanical) adj2 (reperfusion or thrombolysis or revasculari* or recanali*)).mp. or MT.mp. or Endovascular Procedures/ or endovascular.mp. or endo-vascular or EVT.mp. or (reperfusion adj2 (therapy or treatment)).mp. or (exp stroke/su and (reperfusion or revasculari* or recanali*).mp.) |
| Prediction | 5.       Prognosis/ or Forecasting/ or (predict* or prognos* or forecast*).mp or (associat* adj4 (sever* or risk* or outcome* or surviv* or mortality)).mp or determinan*.mp. or exp Comorbidity/ or exp "Severity of Illness Index"/ or (likel* adj5 (sever* or outcome* or surviv* or mortality or associat*)).mp. or biomarker*.mp. |
| Combined Sets | 1. 1 AND 2 AND 3 AND 4 AND 5 |
| **PubMed (**Key Word and MeSH term combination): | |
| Select ‘All Fields’ for each term | |
| Outcome | 1.       (outcome* or “disease course” or followup or follow-up) or "Follow-Up Studies"[Mesh] or "Treatment outcome"[Mesh] or "medical futility"[Mesh] or futil* or "treatment failure"[Mesh] or recover* or progress* or "Glasgow coma scale"[Mesh] or GCS or "Glasgow outcome scale"[Mesh] or GOS or “modified rankin” or mRS or NIHSS or “Barthel scale” or “barthel index” or “Cognition”[Mesh] or cognit* or “Montreal cognitive assessment” or moca or "Quality of Life"[Mesh] or “quality of life” or disab* or dependen* or function* or “Mortality”[Mesh] or mortality or “Death”[Mesh] or death or surviv* or "postoperative complications"[Mesh] or complication* |
| Timing and Setting | 2.       pre-thrombectomy or prethrombectomy or prestroke or pre-stroke or preexisting or pre-existing or premorbid* or pre-morbid* or preintervention or pre-intervention or pre-operative or preoperative or preprocedural or pre-procedural or “before hospital” or “prior to hospital” or pre-hospital or prehospital or admission or preadmission or pre-admission or “emergency assessment” or “early assessment” or “acute assessment” or triag* or “Comorbidity”[Mesh] |
| Population | 1. (“Cerebrovascular accident” or CVA or stroke) or “Stroke”[Mesh] or “brain ischemia”[Mesh] or “brain infarction”[Mesh] or “ischemic stroke”[Mesh] or “cerebral infarction”[Mesh] or “brain infarct” or “brain ischemia” or “brain ischaemia” or “cerebral infarct” or “cerebral ischemia” or “cerebral ischaemia” or “cerebrovascular infarct” or “cerebrovascular ischemia” or “cerebrovascular ischaemia” 2. (“Large vessel occlusion” or LVO or “large artery occlusion” or LAO) or “Thrombectomy”[Mesh] or Thrombectom* or “clot removal” or “clot retrieval” or “clot retriever” or “stent retriever” or stentretriever or “Embolectomy”[Mesh] or embolectomy or “surgical reperfusion” or “surgical revascularisation” or “surgical recanalisation” or “mechanical reperfusion” or “mechanical thrombolysis” or “mechanical revascularisation” or “mechanical recanalisation” or MT or “Endovascular Procedures”[Mesh] or endovascular or endo-vascular or EVT or “reperfusion therapy” or “reperfusion treatment” (stroke/SU[Mesh] and (reperfusion or revasculari* or recanali*)) |
| Prediction | 1. “Prognosis”[Mesh] or “Forecasting”[Mesh] or predict* or prognos* or forecast* or associat* sever* or risk* or outcome* or surviv* or mortality or determinan* or “Comorbidity”[Mesh] or "Severity of Illness Index"[Mesh] or “likely outcome” or likel* or outcome* or surviv* or mortality or biomarker* |
| Combined Sets | 1. 1 AND 2 AND 3 AND 4 AND 5 |
| **Cochrane Library (**Key Word and MeSH term combination): | |
| Use search manager instead of basic for MeSH  Select ‘all text’ for each term | |
| Outcome | 1.       (outcome* or disease course or followup or follow-up) or [mh ^"Follow-Up Studies"] or [mh "Treatment outcome"] or [mh ^"medical futility"] or futil* or [mh ^"treatment failure"] or recover* or progress* or [mh ^"Glasgow coma scale"] or GCS or [mh ^"Glasgow outcome scale"] or GOS or (modified NEAR/2 rankin) or mRS or NIHSS or (Barthel NEAR/2 (scale or index)) or [mh ^Cognition] or cognit* or (Montreal NEAR/2 (cognitive or assessment)) or moca or [mh ^"Quality of Life"] or (quality NEAR/2 life) or disab* or dependen* or function* or [mh ^Mortality] or mortality or [mh ^Death] or death or surviv* or [mh "postoperative complications"] or complication* |
| Timing and Setting | 2.       (pre-thrombectomy or prethrombectomy or prestroke or pre-stroke or preexisting or pre-existing or premorbid* or pre-morbid* or preintervention or pre-intervention or pre-operative or preoperative or preprocedural or pre-procedural or ((before or prior) NEAR/3 (stroke* or thrombectom* or hospital*)) or pre-hospital or prehospital or admission or preadmission or pre-admission or (emergency NEAR/3 assess*) or (early NEAR/3 assess*) or (acute NEAR/3 assess*) or triag*) or [mh Comorbidity] |
| Population | 3.  (Cerebrovascular accident or CVA or stroke) or [mh Stroke] or [mh ^“brain ischemia”] or [mh ^“brain infarction”] or [mh ^“ischemic stroke”] or [mh ^“cerebral infarction”] or ((brain or cerebral or cerebrovascular) NEAR/3 (infarct* or isch?emia))  4.       (Large vessel occlusion or LVO or large artery occlusion or LAO) or [mh Thrombectomy] or Thrombectom* or (clot NEAR/3 (removal or retriev*)) or (stent NEAR/3 retriever) or stentretriever or [mh ^Embolectomy] or embolectomy or ((surgical or mechanical) NEAR/2 (reperfusion or thrombolysis or revasculari* or recanali*)) or MT or [mh ^“Endovascular Procedures”] or endovascular or endo-vascular or EVT or (reperfusion NEAR/2 (therapy or treatment)) or ([mh stroke/SU] and (reperfusion or revasculari* or recanali*)) |
| Prediction | 5.       [mh ^Prognosis] or [mh ^Forecasting] or (predict* or prognos* or forecast*) or (associat* NEAR/4 (sever* or risk* or outcome* or surviv* or mortality)) or determinan* or [mh Comorbidity] or [mh ^"Severity of Illness Index"] or (likel* NEAR/5 (sever* or outcome* or surviv* or mortality or associat*)) or biomarker* |
| Combined Sets | 1. 1 AND 2 AND 3 AND 4 AND 5 |
| **CRD Database (**Key Word and MeSH term combination): | |
| Select ‘any field’ for each term | |
| Outcome | 1.       (outcome* or disease course or followup or follow-up) or MeSH DESCRIPTOR “Follow-Up Studies” EXPLODE ALL TREES or MeSH DESCRIPTOR “Treatment outcome” EXPLODE ALL TREES or MeSH DESCRIPTOR “medical futility” EXPLODE ALL TREES or futil* or MeSH DESCRIPTOR “treatment failure” or recover* or progress* or MeSH DESCRIPTOR “Glasgow coma scale” or GCS or MeSH DESCRIPTOR “Glasgow outcome scale” or GOS or (modified NEAR/2 rankin) or mRS or NIHSS or (Barthel NEAR/2 (scale or index)) or MeSH DESCRIPTOR Cognition or cognit* or (Montreal NEAR/2 (cognitive or assessment)) or moca or MeSH DESCRIPTOR "Quality of Life" or (quality NEAR/2 life) or disab* or dependen* or function* or MeSH DESCRIPTOR Mortality or mortality or MeSH DESCRIPTOR Death or death or surviv* or MeSH DESCRIPTOR “postoperative complications” or complication* |
| Timing and Setting and Population | 2.       ((pre-thrombectomy or prethrombectomy or prestroke or pre-stroke or preexisting or pre-existing or premorbid* or pre-morbid* or preintervention or pre-intervention or pre-operative or preoperative or preprocedural or pre-procedural or ((before or prior) NEAR/3 (stroke* or thrombectom* or hospital*)) or pre-hospital or prehospital or admission or preadmission or pre-admission or (emergency NEAR/3 assess*) or (early NEAR/3 assess*) or (acute NEAR/3 assess*) or triag*) or MeSH DESCRIPTOR Comorbidity) AND (((Cerebrovascular accident or CVA or stroke) or MeSH DESCRIPTOR Stroke EXPLODE ALL TREES or MeSH DESCRIPTOR “brain ischemia” or MeSH DESCRIPTOR “brain infarction” or MeSH DESCRIPTOR “ischemic stroke” or MeSH DESCRIPTOR “cerebral infarction” or ((brain or cerebral or cerebrovascular) NEAR/3 (infarct* or isch?emia))) AND ((Large vessel occlusion or LVO or large artery occlusion or LAO) or MeSH DESCRIPTOR Thrombectomy EXPLODE ALL TREES or Thrombectom* or (clot NEAR/3 (removal or retriev*)) or (stent NEAR/3 retriever) or stentretriever or MeSH DESCRIPTOR Embolectomy or embolectomy or ((surgical or mechanical) NEAR/2 (reperfusion or thrombolysis or revasculari* or recanali*)) or MT or MeSH DESCRIPTOR “Endovascular Procedures”] or endovascular or endo-vascular or EVT or (reperfusion NEAR/2 (therapy or treatment)) or (MeSH DESCRIPTOR stroke/SU and (reperfusion or revasculari* or recanali*)))) |
| Prediction | 3.       MeSH DESCRIPTOR Prognosis or MeSH DESCRIPTOR Forecasting or (predict* or prognos* or forecast*) or (associat* NEAR/4 (sever* or risk* or outcome* or surviv* or mortality)) or determinan* or MeSH DESCRIPTOR Comorbidity] or MeSH DESCRIPTOR "Severity of Illness Index" or (likel* NEAR/5 (sever* or outcome* or surviv* or mortality or associat*)) or biomarker* |
| Combined Sets | 4.       1 AND 2 AND 3 |

Table S2. Study Selection Form

| **Selection criteria for title/abstract/full text screen:** |
| --- |
|  |
| At title/abstract stage - exclude if any evidence the study does NOT meet these criteria |
| At full text stage - exclude if insufficient evidence that the study DOES meet these criteria |
|  |
| **1. STUDY DESIGN** |
| **Quantitative research studies with abstracts published in English from any country with n >2 patients. Observational, interventional, prospective or retrospective prognostic studies.** |
| **1. Exclude:** |
| **Case studies, reviews (including meta-analyses) and protocols. Where multiple studies report on the same prognostic factors within the same cohort (e.g., secondary analyses of the same registry or a republished duplicate of the same study), the most recent study with the largest number of patients and most detail (e.g., conference abstract versus full text) should be retained. Categorical studies which did not at least report patient group proportions and continuous studies which did not report proportions and parametric central tendency and variance for prognostic factor(s) per outcome group.** |
| **2. PARTICIPANTS** |
| **Acute Large Vessel Occlusion (LVO) Ischaemic Stroke patients who undergo mechanical thrombectomy** |
| **2. Exclude:** |
| **Studies examining relevant prognostic factors only within selected samples rather than broad/representative populations (e.g., in particular age groups, within a specific gender, race or stroke severity, or only in patients with failed recanalisation**) **which could bias outcome. Studies which broadly examine ischaemic stroke outcomes and do not report thrombectomy prognosis separately from patients in the sample who did not undergo thrombectomy.** |
| **3. CONCEPT** |
| **3.1. Prognostic Factor(s)** |
| **Physiological Observations (consciousness (GCS/AVPU), blood pressure, heart rate and rhythm, oxygen saturation, respiratory rate, glucose and temperature) routinely collected by or available to non-specialists during the acute prehospital or pre-treatment assessment of thrombectomy patients.** |
| **3.1 Exclude:** |
| **Research studies including factors which are not routinely collected by/available to non-specialists before in-hospital imaging and/or specialist review (e.g., studies including both physiological observations and radiological factors where clinical variables are not reported separately). Studies looking at proxy markers e.g., History of hypertension for current high blood pressure or biomarkers associated with dysregulated physiological observations. Research studies using stroke symptoms or stroke severity, lifestyle factors (smoking, diet, exercise etc.), comorbidities (e.g., hypertension), and demographic factors (age, gender, race etc.) or using models including these factors without reporting them separately from physiological observations. Studies reporting on physiological observations which weren't measured pre-treatment. Studies using non-specific physiological observations with no clear definition (e.g., "Vital Signs"). Research studies report on a factor not used by any other study and therefore unable to be combined in the meta-analysis.** |
| **3.2. Outcome Measure(s)** |
| **Functional dependence at 3 months based on Modified Rankin Score (mRS), dichotomised as favourable (mRS 0-2) and unfavourable (mRS 3-6) functional outcome.** |
| **3.2 Exclude:** |
| **Studies which did not use dichotomised (0-2 versus 3-6) mRS as an outcome measure.** |
| **4. CONTEXT** |
| **Concept is evaluated during standard emergency stroke care (before imaging and thrombectomy).** |
| **4. Exclude:** |
| **Studies involving the use of mobile stroke units or providing thrombectomy treatment outside of current clinical guidelines (e.g., outside of approved standard and extended (advanced imaging) time windows or primarily using first generation thrombectomy devices which are no longer in use, e.g., MERCI retriever)** |
| **If Yes for 1 AND 2 AND 3 AND 4 - INCLUDE** |
| **If No for 1, 2, 3 OR 4 - EXCLUDE (note reason[s] in comments box)** |

| **Study Information** | | | | | | |
| --- | --- | --- | --- | --- | --- | --- |
|  |  |  |  |  |  |  |
| **Study ID** | **Author** | **Year of Publication** | **Title** | **Country of Origin** | **Same cohort as another included study?** (say which then exclude secondary study or least detailed study unless there is added value in both) | **Subsequently excluded after realising ineligible (Yes/No)** |

Table S3. Data Extraction Form

| **Method** | | | | | | | | | | | | | | | | | | | | | |
| --- | --- | --- | --- | --- | --- | --- | --- | --- | --- | --- | --- | --- | --- | --- | --- | --- | --- | --- | --- | --- | --- |
| **Design Information** | | | | | | | | | | | | | | | | | | | | | |
| **Research Method** | | **Study Design** | | **Study Setting** | **Patient Inclusion Criteria** | | **Patient Exclusion Criteria** | | **Vascular Territory** | **Prognostic Factor(s)** | | | **Prognostic factor measurem-ent timing (primary timepoint)** | | **Outcome Measure (s) (e.g mRS; NIHSS; Infarct Volume; MMCAI)** | | | | **Timing of Outcome Measure (primary outcome)** | | **Outcome assessor blinded to baseline prognosis?** |
| Qualitati-ve | | Cross-sectional | | Primary stroke unit |  | |  | | Anterior |  | | | <4.5h | |  | | | | <24h | | Yes |
| Quantit-ative | | Case-control | | Comprehensive centre |  |  |  |  | Posterior |  |  |  | <6h | |  |  |  |  | <48h | | No |
| Mixed | | Cohort | | Tertiary hospital |  |  |  |  | Mix |  |  |  | <12h | |  |  |  |  | <72h | | Unclear |
| Unclear | | Randomi-sed Controlled Trial | | Ambulance/prehospital |  |  |  |  | Unclear |  |  |  | Admission | |  |  |  |  | Discharge | | Not Reported |
|  | | Meta analysis | | Cohort and RCT |  |  |  |  | Not Reported |  |  |  | Unclear | |  |  |  |  | 1 Week | |  |
|  |  | Unclear | | Unclear |  |  |  |  |  |  |  |  | Not Reported | |  |  |  |  | 1 month | |  |
|  |  | Not Reported | | Not Reported |  |  |  |  |  |  |  |  |  |  |  |  |  |  | 3 months | |  |
|  |  |  |  |  |  |  |  |  |  |  |  |  |  |  |  |  |  |  | 6 months | |  |
|  |  |  |  |  |  |  |  |  |  |  |  |  |  |  |  |  |  |  | 1 year | |  |
|  |  |  |  |  |  |  |  |  |  |  |  |  |  |  |  |  |  |  | >1year | |  |
|  |  |  |  |  |  |  |  |  |  |  |  |  |  |  |  |  |  |  | Unclear | |  |
|  | |  | |  |  | |  | |  |  | | |  | |  | | | | Not Reported | |  |
| **Method** | | | | | | | | | | | | | | | | | | | | |  |
| **Clinical Treatment Context** | | | | | | | | | | | | | | | | | | | | |  |
| **Thrombectomy technique** | | | | | | | | | | **Thrombectomy within usual time window (~6-7h) or extended time window (salvageable tissue on advanced imaging up to 24h)** | | | | | | | **Proportion received bridging thrombolysis (enter % OR =proportion/total sample)** | | | |  |
| Aspiration-catheter | | | | | | | | | | Standard | | | | | | |  | | | |  |
| Stent-retriever | | | | | | | | | | Extended | | | | | | |  |  |  |  |  |
| Primary Combined Approach (stent-retriever and aspiration-catheter) | | | | | | | | | | Unclear | | | | | | |  |  |  |  |  |
| Aspiration-catheter with balloon catheter | | | | | | | | | | Not Reported | | | | | | |  |  |  |  |  |
| Stent-retriever with balloon catheter | | | | | | | | | |  |  |  |  |  |  |  |  |  |  |  |  |
| Primary Combined Approach (stent-retriever and aspiration-catheter) with balloon catheter | | | | | | | | | |  |  |  |  |  |  |  |  |  |  |  |  |
| Mix | | | | | | | | | |  |  |  |  |  |  |  |  |  |  |  |  |
| Unclear | | | | | | | | | |  |  |  |  |  |  |  |  |  |  |  |  |
| Not Reported | | | | | | | | | |  |  |  |  |  |  |  |  |  |  |  |  |
| **Results** | | | | | | | | | | | | | | | | | | | | |  |
| **Sample** | | **Model/Score** | | | **Physiological Observations**  **[Note: Each of these factors has its own columns in the extraction spreadsheet]** | | | | | | | | | | | | | | | |  |
| **Total sample size** (and thrombectomy sample size if study had a wider sample) | | **Overall model/score prognostic results if applicable** (e.g. prognostic accuracy if model only included eligible factors) | | | **Glucose** **Consciousness/GCS Heart Rate**  **Systolic Blood Pressure**  **Diastolic Blood Pressure**  **Atrial Fibrillation** **Peripheral Oxygen Saturation/Spo2** **Respiration Rate** **Temperature**  (for continuous data: yes/no only, for categorical/non-parametric data: state whether Nominal or Ordinal and chosen threshold if applicable) | | | | | **Number of patients with result reported** | | | **% of sample with data for this factor** | | | **Type of Continuous Data** | | | **Result(s)** (P-value, OR, etc... and include direction of effect) and mean and standard deviation/variance of measurement (if applicable) | |  |
|  | |  | | |  | | | | |  | | |  | | | Interval | | |  | |  |
|  |  |  |  |  |  |  |  |  |  |  |  |  |  |  |  | Ratio | | |  |  |  |
|  |  |  |  |  |  |  |  |  |  |  |  |  |  |  |  | Unclear | | |  |  |  |
|  |  |  |  |  |  |  |  |  |  |  |  |  |  |  |  | Not Reported | | |  |  |  |
|  |  |  |  |  |  |  |  |  |  |  |  |  |  |  |  | Not Applicable | | |  |  |  |
| **Results** | | | | | | | | | | | | | | | | | | | | |  |
| **Missing Data** | | | | | | | | | | | | | | | | | | | | |  |
| **Patients missing from analysis?** | | | | | | | **Explanation for patients missing from analysis?** | | | | | | | | **Explanation (comment)** | | | | | |  |
| Yes | | | | | | | Yes | | | | | | | |  | | | | | |  |
| No | | | | | | | No | | | | | | | |  |  |  |  |  |  |  |
| Unclear | | | | | | | Unclear | | | | | | | |  |  |  |  |  |  |  |
| Not Reported | | | | | | | Not Reported | | | | | | | |  |  |  |  |  |  |  |
| **Quality** | | | | | | | | | | | | | | | | | | | | |  |
| **Quality Score** | | | | | | | | | | | **Risk of bias** | | | | | | | | | |  |
| /14 | | | | | | | | | | | Low | | | | | | | | | |  |
|  |  |  |  |  |  |  |  |  |  |  | Medium | | | | | | | | | |  |
|  |  |  |  |  |  |  |  |  |  |  | High | | | | | | | | | |  |

Table S4. Quality Assessment (Modified QUIPS Tool)

| **1. Study Participation (Goal: To judge the risk of selection bias (likelihood that relationship between PF and outcome is different for participants and eligible non-participants)).** | | | | | | | | | | | | | | | | | | | | | | | | | | |  |
| --- | --- | --- | --- | --- | --- | --- | --- | --- | --- | --- | --- | --- | --- | --- | --- | --- | --- | --- | --- | --- | --- | --- | --- | --- | --- | --- | --- |
| **Source of target population** | | **Inclusion and Exclusion Criteria** | | | | | | **Adequate study participation** | | **Baseline characteristics** | | | | | **Summary Study Participation** | | | | | | | | | | | |  |
| **The source population, sampling method (including methods to limit bias) and sampling setting are adequately described** | | **Inclusion and Exclusion Criteria are adequately described (e.g., explicit diagnostic criteria and time window) and likely to represent the population of interest/unlikely to introduce inappropriate bias** | | | | | | **There is adequate participation in the study by eligible individuals (100-300 = Partial)** | | **The baseline study sample (i.e., individuals entered into the study) is adequately described for key characteristics (N, Age, Genders, Obs/Comorbidities, Stroke type & Reperfusion treatment type)** | | | | | **The study sample represents the population of interest on key characteristics, sufficient to limit potential bias of the observed relationship between prognostic factor and outcome.** | | | | | **Risk of bias**  (Automatic calculation) | | | | | **Comment(s) if applicable (Red fill if you need to come back to it)** | |  |
| Yes (1) | | Yes (1) | | | | | | Yes (1) | | Yes (1) | | | | | Automatic calculation (/4) | | | | | High (0-1) | | | | |  | |  |
| Partial (0.5) | | Partial (0.5) | | | | | | Partial (0.5) | | Partial (0.5) | | | | |  |  |  |  |  | Moderate (1.5-2.5) | | | | |  |  |  |
| No (0) | | No (0) | | | | | | No (0) | | No (0) | | | | |  |  |  |  |  | Low (3-4) | | | | |  |  |  |
| Unsure (0) | | Unsure (0) | | | | | | Unsure (0) | | Unsure (0) | | | | |  |  |  |  |  |  |  |  |  |  |  |  |  |
| **2. Study Attrition (Goal: To judge the risk of attrition bias (likelihood that relationship between PF and outcome are different for completing and non-completing participants))** | | | | | | | | | | | | | | | | | | | | | | | | | | |  |
| **Reasons and potential impact of subjects lost to follow-up** | | | **Outcome and prognostic factor information on those lost to follow-up** | | | | | | **Study Attrition Summary** | | | | | | | | | | | | | | | | | |  |
| **Reasons for loss to follow-up are provided. 'Yes' if clear none were lost.** | | | **Participants lost to follow-up are adequately described for key characteristics (N, age, gender, reperfusion treatment(s), reperfusion success/mortality/acute outcomes if applicable) - Yes if clear none lost** | | | | | | **Loss to follow-up (from baseline sample to study population analysed) is not associated with key characteristics (i.e., the study data adequately represent the sample) sufficient to limit potential bias to the observed relationship between prognostic factor and outcome.** | | | | | | | **Risk of bias** (Automatic calculation) | | | | | | | **Comment(s) if applicable (Red fill if you need to come back to it)** | | | |  |
| Yes (1) | | | Yes (1) | | | | | | Automatic calculation (/2) | | | | | | | High (0-0.5) | | | | | | |  | | | |  |
| Partial (0.5) | | | Partial (0.5) | | | | | |  |  |  |  |  |  |  | Moderate (1) | | | | | | |  |  |  |  |  |
| No (0) | | | No (0) | | | | | |  |  |  |  |  |  |  | Low (1.5-2) | | | | | | |  |  |  |  |  |
| Unsure (0) | | | Unsure (0) | | | | | |  |  |  |  |  |  |  |  |  |  |  |  |  |  |  |  |  |  |  |
| **3. Prognostic Factor Measurement (Goal: To judge the risk of measurement bias related to how PF was measured (differential measurement of PF related to the level of outcome))** | | | | | | | | | | | | | | | | | | | | | | | | | | |  |
| **Definition of the prognostic factor** | | | | | | | **Valid and reliable measurement of the prognostic factor** | | | | | | | **Prognostic Factor Measurement Summary** | | | | | | | | | | | | |  |
| **A clear definition or description of prognostic factor(s) and the time point of measurement is provided (e.g., including thresholds or durations and clear specification of the method of measurement).** | | | | | | | **Method of Prognostic factor measurement is adequately valid and reliable to limit misclassification bias (e.g., information from medical notes and limited reliance on recall)** | | | | | | | **Prognostic factor is adequately measured in study participants to sufficiently limit potential bias** | | | | **Risk of bias** (Automatic calculation) | | | | | **Comment(s) if applicable (Red fill if you need to come back to it)** | | | |  |
| Yes (1) | | | | | | | Yes (1) | | | | | | | Automatic calculation (/2) | | | | High (0-0.5) | | | | |  | | | |  |
| Partial (0.5) | | | | | | | Partial (0.5) | | | | | | |  |  |  |  | Moderate (1) | | | | |  |  |  |  |  |
| No (0) | | | | | | | No (0) | | | | | | |  |  |  |  | Low (1.5-2) | | | | |  |  |  |  |  |
| Unsure (0) | | | | | | | Unsure (0) | | | | | | |  |  |  |  |  |  |  |  |  |  |  |  |  |  |
| **4. Outcome Measure (Goal: To judge the risk of bias related to the measurement of outcome (differential measurement of outcome related to the baseline level of PF))** | | | | | | | | | | | | | | | | | | | | | | | | | | |  |
| **Definition of the Outcome** | | | | **Valid and reliable measurement of outcome** | | | | | | | | **Outcome Measurement Summary** | | | | | | | | | | | | | | |  |
| **A clear definition of outcome is provided, including duration of follow-up and level and extent of the outcome construct.** | | | | **The method of outcome measurement used is adequately valid and reliable to limit misclassification bias (e.g., blind measurement (DE cell O3), qualified assessors, adjudication and confirmation of outcome with valid and reliable test and valid threshold(s) if applicable). 'Partial' if not clear blinded but otherwise valid and reliable.** | | | | | | | | **Outcome of interest is adequately measured in study participants to sufficiently limit potential bias.** | | | | | | | **Risk of bias** (Automatic calculation) | | | | | **Comment(s) if applicable (Red fill if you need to come back to it)** | | |  |
| Yes (1) | | | | Yes (1) | | | | | | | | Automatic calculation (/2) | | | | | | | High (0-0.5) | | | | |  | | |  |
| Partial (0.5) | | | | Partial (0.5) | | | | | | | |  |  |  |  |  |  |  | Moderate (1) | | | | |  |  |  |  |
| No (0) | | | | No (0) | | | | | | | |  |  |  |  |  |  |  | Low (1.5-2) | | | | |  |  |  |  |
| Unsure (0) | | | | Unsure (0) | | | | | | | |  |  |  |  |  |  |  |  |  |  |  |  |  |  |  |  |
| **5. Study Confounding (Goal: To judge the risk of bias due to confounding (i.e., the effect of prognostic factor is distorted by another factor that is related to prognostic factor and outcome).)** | | | | | | | | | | | | | | | | | | | | | | | | | | |  |
| **Appropriate accounting for confounding** | | | | | | | | | | | **Study Confounding Summary** | | | | | | | | | | | | | | | |  |
| **Important potential confounders are accounted for in the study design (e.g., matching for key variables, stratification, or initial assembly of comparable groups).** | | | | | | **Important potential confounders are accounted for in the analysis (i.e., appropriate adjustment). Partial for analysis including confounds without adjustment.** | | | | | **Important potential confounders are appropriately accounted for, limiting potential bias with respect to the relationship between prognostic factor(s) and outcome.** | | | | | | | | | | **Risk of bias** (Automatic calculation) | | | | | **Comment(s) if applicable (Red fill if you need to come back to it)** |  |
| Yes (1) | | | | | | Yes (1) | | | | | Automatic calculation (/2) | | | | | | | | | | High (0-0.5) | | | | |  |  |
| Partial (0.5) | | | | | | Partial (0.5) | | | | |  |  |  |  |  |  |  |  |  |  | Moderate (1) | | | | |  |  |
| No (0) | | | | | | No (0) | | | | |  |  |  |  |  |  |  |  |  |  | Low (1.5-2) | | | | |  |  |
| Unsure (0) | | | | | | Unsure (0) | | | | |  |  |  |  |  |  |  |  |  |  |  |  |  |  |  |  |  |
| **6. Statistical Analysis and Reporting (Goal: To judge the risk of bias related to the statistical analysis and presentation of results.)** | | | | | | | | | | | | | | | | | **Overall Risk of Bias** | | | | | | | | | |  |
| **Presentation of analytical strategy** | | | | | **Statistical Analysis and Presentation Summary** | | | | | | | | | | | |  | | | | |  | | | | |  |
| **There is sufficient presentation of data to assess the adequacy of the analysis.** | **The selected statistical method is adequate for the design of the study.** | | | | **The selected statistical method is adequate for the design of the study, limiting potential for presentation of invalid or spurious results.** | | | | | **Risk of Bias** | | | **Comment(s) if applicable (Red fill if you need to come back to it)** | | | | **Quality Score** | | | | | **Risk of Bias** | | | | |  |
| Yes (1) | Yes (1) | | | | Automatic calculation (/2) | | | | | High (0-0.5) | | |  | | | | Automatic calculation **(\14)** | | | | | High (0-3.5) | | | | |  |
| Partial (0.5) | Partial (0.5) | | | |  |  |  |  |  | Moderate (1) | | |  |  |  |  |  |  |  |  |  | Moderate (4-10) | | | | |  |
| No (0) | No (0) | | | |  |  |  |  |  | Low (1.5-2) | | |  |  |  |  |  |  |  |  |  | Low (10.5-14) | | | | |  |
| Unsure (0) | Unsure (0) | | | |  |  |  |  |  |  |  |  |  |  |  |  |  |  |  |  |  |  |  |  |  |  |  |

| **Risk of Bias Scoring**  Yes = 1  Partial = 0.5  No/Unsure = 0 | | | |  |
| --- | --- | --- | --- | --- |
| For 2 items: | | For 4 items: | |  |
| 0 | High | 0 | High |  |
| 0.5 | High | 0.5 | High |  |
| 1 | Moderate | 1 | High |  |
| 1.5 | Low | 1.5 | Moderate |  |
| 2 | Low | 2 | Moderate |  |
|  |  | 2.5 | Moderate |  |
|  |  | 3 | Low |  |
|  |  | 3.5 | Low |  |
|  |  | 4 | Low |  |
| Overall Total: | | | |  |
| 0-3.5 High Risk | | | |  |
| 4-10 Moderate Risk | | | |  |
| 10.5-14 Low Risk | | | |  |

Table S5. Included Pre-treatment Prognostic Factors and Post-treatment Outcome Measures for Thrombectomy

|  |  | **Post-Thrombectomy Outcome Measures** | | | | | | | |
| --- | --- | --- | --- | --- | --- | --- | --- | --- | --- |
|  | | **modified Rankin Score (mRS)** | | | **Other Outcome Measures** | | | | **Totals** |
|  |  | **0-2 versus 3-6** | **0-1 versus 2-6** | **Ordinal mRS** | **Disability (other)** | **Mortality** | **Neurological** | **Radiological** |  |
| **Physiological Observations** | **Glucose (N=24)** | **24** | **3** | **3** | **1** | **11** | **1** | **0** | **43** |
|  | **Systolic Blood Pressure (N=16)** | **16** | **0** | **0** | **0** | **4** | **0** | **1** | **21** |
|  | **Diastolic Blood Pressure (N=13)** | **13** | **0** | **0** | **0** | **4** | **0** | **1** | **18** |
|  | **ECG Atrial Fibrillation (N=3)** | **3** | **0** | **0** | **0** | **2** | **0** | **0** | **5** |
|  | **Consciousness (GCS) (N=2)** | **2** | **0** | **0** | **0** | **0** | **0** | **0** | **2** |
|  | **Totals** | **58** | **3** | **3** | **1** | **21** | **1** | **2** | **89** |

Table S6. Pre-treatment Glucose for Predicting Outcome after Thrombectomy

| **Author(s)** | **Number of patients (%)** | **Prognostic Factor Continuous/Categorical Data Type** | **Prognostic Factor Threshold** | **Modified Rankin (mRS) 0-2 versus 3-6 Results** | **Other Outcome Measures(s) (Data type)** | **Other Outcome Measure Timepoint(s)** | **Other Outcome Measure Results** | **Risk of Bias /14** |
| --- | --- | --- | --- | --- | --- | --- | --- | --- |
| Shriki et al. (2020) | 134/134 (100%) | Ratio/N/A | N/A | No significant difference in serum glucose in the favourable (mRS 0-2: 53/134- 40%, mean 145mg/dL, SD 70mg/dL) versus unfavourable (mRS 3-6: 81/134- 60%, mean 134mg/dL, SD 43mg/dL) functional outcome group, p=0.33. | N/A | N/A | N/A | 12.5/14-Low |
| Ozdemir et al. (2015) | 70/70 (100%) | Ratio/N/A | N/A | Significantly higher serum glucose in the unfavourable (mRS 3-6: 37/70-53%, mean 187mg/dL, SD 67.6mg/dL) versus favourable (mRS 0-2: 33/70-47%, mean 126mg/dL, SD 380.5mg/dL) functional outcome group, p=0.002. Higher serum glucose significantly associated with lower odds of favourable (mRS 0-2) functional outcome (OR 0.098, 95% CI 0.0962-0.996), p=0.017. | N/A | N/A | N/A | 11/14-Low |
| Broocks, G., et al., 2020Broocks et al. (2020) | 178/178 (100%) | Ratio/N/A | N/A | Significantly higher blood glucose in the unfavourable (mRS 3-6: 109/178- 61%, mean 141mg/dL, SD 41mg/dL) versus favourable (mRS 0-2: 69/178- 39%, mean 117mg/dL, SD 25mg/dL) functional outcome group, P<0.01. Significantly lower odds of a favourable (mRS 0-2) functional outcome per 1 mg/dl glucose increase (univariate OR 0.097, 95%CI 0.096–.99, P<0.001, multivariate OR 0.097, 95%CI 0.096–.99, P<0.001). | N/A | N/A | N/A | 13/14-Low |
| Zi, W., et al., 2017Zi et al. (2017) | 649/698 (92.98%) | Ratio/N/A | N/A | Significantly higher blood glucose (N=649/698- 93%) in the unfavourable (mRS 3-6: 394/698- 56%, mean 8.3mmol/L, SD 3.9mmol/L) versus favourable (mRS 0-2: 304/698- 44%, mean 6.9mmol/L, SD 2.5mmol/L) functional outcome group, P<0.001. Significant association between higher glucose and unfavourable (mRS 3-6) functional outcome (Multivariate OR 1.09, 95% CI 1.01–1.18), p= 0.002 (missing 3-month data substituted with discharge mRS but statistics the same for patients with 3-month mRS only (N=650/698- 93%)). | N/A | N/A | N/A | 13.5/14-Low |
| Gordon et al. (2018) | 79/79 (100%) | Ratio/N/A | N/A | No significant difference in blood glucose in the favourable (mRS 0-2: 35/79- 44%, mean 143.71mg/dL, SD 530.52mg/dL) versus unfavourable (mRS 3-6: 44/79- 56%, mean 161.68mg/dL, SD 74.65mg/dL) functional outcome group, p=0.0234. | N/A | N/A | N/A | 10/14-Moderate |
| Suissa et al. (2020) | 41/41 (100%) | Ratio/N/A | N/A | Significantly higher blood glucose in the unfavourable (mRS 3-6: 23/41- 56%, mean 142mg/dL, SD 38mg/dL) versus favourable (mRS 0-2: 18/41- 44%, mean= 125mg/dL, SD 21mg/dL) functional outcome group, p=0.0378. | N/A | N/A | N/A | 10/14-Moderate |
| Shi et al. (2014) | 109/109 (100%) | Ratio/N/A | N/A | Significantly higher blood glucose in patients with unfavourable (mRS 3-6: 53/109- 49%, mean 159mg/dL, SD 68mg/dL) versus favourable (mRS 0-2: 56/109- 51%, mean 113mg/dL, SD 28mg/dL) functional outcome, p=0.0010. | N/A | N/A | N/A | 12/14-Low |
| Sallustio et al. (2019) | 270/270 (100%) | Ratio/N/A | N/A | Significantly higher glucose in the unfavourable (mRS 3-6: 168/270-62%, mean 143.67mg/dL, SD 570.5mg/dL) versus favourable (mRS 0-2: 102/270-37%, mean 118.18mg/dL, SD 35.0mg/dL) functional outcome group, p=0.001. | N/A | N/A | N/A | 12.5/14-Low |
| Karamchandani et al. (2022) | 57/57 (100%) | Ratio/N/A | N/A | No significant difference in blood glucose in the favourable (mRS 0-2: 19/57- 33%, mean 164mg/dL, SD 67.09mg/dL) versus unfavourable (mRS 3-6: 38/57- 67%, mean 155.79mg/dL, SD 55.11mg/dL) functional outcome group, p=0.886. | N/A | N/A | N/A | 12/14-Low |
| Phuong et al. (2022b) | 49/49 (100%) | Ratio/N/A | N/A | No significant difference in glucose in the favourable (mRS 0-2: 22/49- 45%, mean 8.3mmol/L, SD 2.6mmol/L) versus unfavourable (mRS 3-6: 27/49- 55%, mean 9.7mmol/L, SD 4.9mmol/L) functional outcome group, P>0.05. | N/A | N/A | N/A | 10/14-Moderate |
| Nisar et al. (2021) | 188/188 (100%) | Ratio/N/A | N/A | Significantly higher blood glucose in patients with unfavourable (mRS 3-6: 134/188- 71%, mean 153.05mg/dL, SD 70.57mg/dL) versus favourable (mRS 0-2: 54/188- 29%, mean 123.78mg/dL, SD 32.31mg/dL) functional outcome (95% CI 44.1-14.44), P<0.001. | Mortality and Survival (Binary) | 3 months | Significantly higher glucose in the Mortality (n=58, mean 59.95, SD 75) versus Survival (n=130, mean 137.81, SD 56.4) group (95%CI 44.05-0.24), p=0.048. | 12.5/14-Low |
| Jiang et al. (2015) | 89/89 (100%) | Ratio/N/A | N/A | Significantly higher serum glucose with unfavourable (mRS 3-6: 52/89- 58%, mean 9.43mmol/L, SD 4.41mmol/L) versus favourable (mRS 0-2: 37/89- 42%, mean 60.53mmol/L, SD 2.38mmol/L) functional outcome (Univariate OR 1.37, 95% CI 1.13-1.66, Multivariate OR 1.31, 95% CI 1.06-1.63). | Mortality and Survival (Binary) | 3 months | Significantly higher glucose in the mortality (N=21/89- 24%, mean 1.26mmol/L, SD 4.33mmol/L) versus survival (N=68/89- 76%, mean 70.58mmol/L, SD 3.63mmol/L) group (OR 1.17, 95% CI 1.03 - 1.33) (no p-value). Not significant in the multivariate analysis. | 12.5/14-Low |
| Cao et al. (2021) | 101/101 (100%) | Ratio/N/A | N/A | No significant difference in glucose in the favourable (mRS 0-2: 50/101- 50%, mean 7.84mmol/L, SD 2.83mmol/L) versus unfavourable (mRS 3-6: 51/101- 50%, mean 8.76mmol/L, SD 3.08mmol/L) functional outcome group, p=0.065. | Mortality and Survival (Binary) | 3 months | Significantly higher glucose in the mortality (N=27/101- 27%, mean 9.2mmol/L, SD 3.4mmol/L) versus survival (N=74/101- 74%, mean 7.97mmol/L, SD 2.76mmol/L) group, p=0.049. | 12.5/14-Low |
| Sun et al. (2021) | 212/212 (100%) | Ratio/N/A | N/A | Significantly higher admission blood glucose in the unfavourable (mRS 3-6: 98/212- 46%, mean 7.83, SD 2.90) versus favourable (mRS 0-2: 114/212-54%, mean 6.26, SD 1.91) functional outcome group, P<0.001. Significant association between higher glucose and unfavourable (mRS 3-6) functional outcome (multivariate OR 1.32, 95%CI 1.13-10.55), p=0.001. | Mortality and Survival (Binary) | 3 months | Significantly higher glucose in the mortality (N=31/212 -15%, mean 7.94mmol/L, SD 3.05mmol/L) versus survival (N=181/212-85%, mean 6.83mmol/L, SD 2.42mmol/L) group (OR 1.14, 95% CI 0.099-1.30), p=0.026. No significant association between glucose and survival (OR 1.14, 95%CI 0.099-130), p=0.068. | 9.5/14-Moderate |
| Huo et al. (2016) | 36/36 (100%) | Ratio/N/A | N/A | No significant difference in glucose in the unfavourable (mRS 3-6: 26/36- 72%, mean 10.71mmol/L, SD 5.09mmol/L) versus favourable (mRS 0-2: 10/36- 28%, mean 7.43mmol/L, SD 3.43mmol/L) functional outcome group, p=0.070. No significant association between glucose and unfavourable functional outcome (OR 1.29, 95%CI 0.096-1.73). | Mortality and Survival (Binary) | 3 months | No significant association between glucose and mortality (N= 11/36- 31%, mean 11.98mmol/L, SD 6.31mmol/L) versus survival (N=25/36- 69%, mean 8.84mmol/L, SD 5.95-11.12mmol/L) (OR 1.14, 95%CI 0.098-1.33), p=0.074. | 12.5/14-Low |
| Wu et al. (2019) | 118/118 (100%) | Ratio/Binary | AUC <7.2mmol/L versus >/=7.2mmol/L | Significantly higher blood glucose in the unfavourable (mRS 3-6: 71/118 - 60%, mean 9.6mmol/L, SD 4.6mmol/L, median 8.1, IQR 3.2) versus favourable (mRS 0-2: 47/118- 40%, mean 7.1mmol/L, SD 1.9mmol/L, median 6.6mmol/L, IQR 2.3) functional outcome group, t/Z=3.93, P<0.01. Higher glucose significantly associated with less favourable functional outcome (ordinal mRS) (Beta 0.0421, SE 0.0153, Wald 7.602, p=0.006, OR 10.524, 95%CI 1.129-2.055) and unfavourable (mRS 3-6) functional outcome (AUC 0.0714, Threshold 7.2mmol/L, p <.001, 95%CI 0.0620-0.809, Sensitivity 73.2%, Specificity 61.7%). | N/A | N/A | N/A | 12/14-Low |
| Chen et al. (2020) | 248/248 (100%) | Ratio/Binary | Elevated blood glucose (not defined), not-elevated blood glucose (not defined) | Significantly higher blood glucose in the unfavourable (mRS 3-6: 131/248- 53%, mean 8.3mmol/L, SD 3.1mmol/L) versus favourable (mRS 0-2: 117/248- 47%, mean 8.0mmol/L, SD 3.2mmol/L) functional outcome group, p=0.424. Elevated blood glucose (N=194) was significantly greater in the unfavourable (mRS 3-6: 113/194- 69%) versus favourable (N=81/194-42%) functional outcome group, p=0.001. | N/A | N/A | N/A | 13/14-Low |
| Kim et al. (2018) | 296/309 (95.79%) | Ratio/Binary | Hyperglycaemia (>140 mg/dL) versus No hyperglycaemia (</=140 mg/dL) | Significantly higher glucose in the unfavourable (mRS 3-6: 144/296- 49%, mean 140mg/dl, SD 62mg/dL) versus favourable (mRS 0-2: 152/296- 51%, mean 124mg/dL, SD, 47mg/dL) functional outcome group, p=0.01. Significant association between lower glucose and favourable (mRS 0-2) functional outcome (uOR 0.094, 95% CI 0.090-0.99), p=0.02, (aOR 0.095, 95% CI 0.089-1.00), p=0.06. | Excellent (mRS 0-1) and Non-excellent (mRS 2-6) Functional Outcome (Binary); Ordinal mRS; Mortality and Survival (Binary) | 3 months | Significantly higher glucose in the less favourable (mRS 2-6: 191/296- 65%, mean 138 mg/dL, SD 60mg/dL) versus excellent (mRS 0-1: N=105/296- 36%, mean 120mg/dl, SD, 43mg/dL) functional outcome group, p=0.008. Significant association between lower glucose and excellent (mRS 0-1) functional outcome (uOR 0.093, 95% CI 0.087-0.98), p=0.009 but not when adjusted for other factors (aOR 0.095, 95% CI 0.089-1.01), p=0.1. Significant association between increasing glucose levels (per 10mg/dl increase) less favourable functional outcomes (ordinal mRS) (uOR 1.05, 95%CI 1.01–1.09), p=0.015, but not in adjusted analyses (aOR 1.03, 95% CI 0.099–1.07), p=0.11. Hyperglycaemia (>140 mg/dL) associated with unfavourable outcome (ordinal mRS) compared with normoglycaemia/hypoglycaemia (≤140 mg/dl) (uOR 0.53, 95% CI 0.034–.83), p=0.005, (aOR 0.064, 95% CI 0.040–1.03), p=0.07. No significant difference in glucose in the mortality (N=44/309- 14%, mean 136mg/dL, SD 49mg/dL) versus survival (N=265/309- 86%, mean 130mg/dL, SD 56mg/dL) group, p=0.5. No significant association between mean glucose and mortality (uOR 1.02, 95% CI 0.097-1.07), p=0.50 (aOR 0.099, 95% CI 0.093-1.06), p=0.8. | 11.5/14-Low |
| Huo et al. (2019) | 149/149 (100%) | Ratio/Binary | Hyperglycaemia (≥7.8 mmol/L) versus No hyperglycaemia (<7.8mmol/L) | Significantly higher serum glucose in patients with unfavourable (mRS 3-6: 66/149 - 44.3%, mean 8.19mmol/L, SD 3.27mmol/L) versus favourable (mRS 0-2: 83/149- 55.7%, mean 6.49mmol/L, SD 1.45mmol/L) functional outcome, P<0.001. Significantly lower odds of a favourable (mRS 0-2) functional outcome with higher glucose (uOR 0.070, 95% CI 0.57–.85), P<0.001, (aOR 0.017, 95% CI 0.006–.45), P<0.001. A greater proportion of normoglycaemia patients (Glucose <7.8mmol/L: 133/149- 82%) had favourable (mRS 0-2: 75/113- 66%) versus unfavourable (mRS 3-6: 38/113- 34%) functional outcome compared with hyperglycaemia (Glucose >/=7.8mmol/L: 36/149- 24%) favourable (mRS 0-2: 8/36- 22%) versus unfavourable (mRS 3-6: 28/36- 78%) functional outcome. Significantly lower odds of favourable functional outcome (mRS 0-2) with hyperglycaemia (uOR 0.015, 95%CI 0.006–.35), P<0.001, (aOR 0.075, 95%CI 0.061–.92), p=0.005. Significantly lower odds of favourable (mRS 0-2) functional outcome with hyperglycaemia (uOR 0.015, 95%CI 0.006–.35), P<0.001, (aOR 0.075, 95%CI 0.061–.92), p=0.005. | Excellent (mRS 0-1) and Non-excellent (mRS 2-6) Functional Outcome (Binary); Mortality and Survival (Binary); Favourable (Barthel Index (BI) score 95–100) and Unfavourable (BI 0-95) Functional Outcome (Binary); Dramatic Neurological Improvement (DrNI: NIHSS reduction or NIHSS 0-2) and No DrNI (No NIHSS reduction or NIHSS >2) (Binary) | 24h (NIHSS) and 3 months (mRS, BI and Mortality) | Significantly higher glucose in patients with non-excellent (mRS 2-6: 85/149 - 57%, mean 7.86mmol/L, SD 2.97mmol/L) versus excellent (mRS 0-1: 64/149- 43%, mean 6.43mmol/L, SD 1.6mmol/L) functional outcome, P<0.001. Significantly lower odds of an excellent (mRS 0-1) functional outcome with higher glucose (uOR 0.072, 95% CI 0.58–.89), p=0.002, (aOR 0.023, 95% CI 0.008–.64), p=0.005. A greater proportion of normoglycaemia patients (Glucose <7.8mmol/L: 133/149- 82%) had excellent (mRS 0-1: 58/113- 51%) versus non-excellent (mRS 2-6: 55/113- 49%) functional outcome compared with hyperglycaemia (Glucose >/=7.8mmol/L: 36/149- 24%) excellent (mRS 0-1: 6/36- 17%) versus non-excellent (mRS 2-6: 30/36- 83%) functional outcome. Significantly lower odds of excellent (mRS 0-1) functional outcome with hyperglycaemia (uOR 0.019, 95%CI 0.007–.49), P<0.001, (aOR 0.077, 95%CI 0.062–.95), p=0.017. No significant difference in glucose in the DrNI (N=62/149- 42%, mean 6.85mmol/L, SD 1.95mmol/L) versus No DrNI (N=87/149- 58%, mean 70.53mmol/L, SD 2.91mmol/L) group, p=0.11. No significant association between glucose and DrNI (uOR 0.089, 95%CI 0.077-1.03), p=0.119, (aOR 0.54, 95%CI 0.022-1.33), p=0.182. A greater proportion of normoglycaemia patients (Glucose <7.8mmol/L: 133/149- 82%) had DrNI (N=52/113- 46%) versus No DrNI (N=61/113- 54%) compared with hyperglycaemia (Glucose >/=7.8mmol/L: 36/149- 24%) DrNI (N=10/36- 28%) versus No DrNI (N=26/36- 72%). No significant association between DrNI and hyperglycaemia (uOR 0.045, 95%CI 0.020–1.02), p=0.053, (aOR 0.093, 95%CI 0.079–1.09), p=0.376. Significantly higher glucose in the unfavourable (BI <95: 79/149- 53%, Mean 7.89mmol/L, SD 3.09mmol/L) versus favourable (BI 95–100: 70/149- 47%, Mean 60.51mmol/L, SD 10.54mmol/L) functional outcome group, P<0.001. Significant association between higher glucose and lower odds of a favourable (BI 95-100) outcome (uOR 0.074, 95%CI 0.061–.90), p=0.003, (aOR 0.030, 95%CI 0.012–.79), p= 0.0014. A greater proportion of normoglycaemia patients (Glucose <7.8mmol/L: 133/149- 82%) had favourable functional outcome (BI 95-100: 62/113- 55%) versus unfavourable functional outcome (BI <95: 51/113- 45%) compared with hyperglycaemia (Glucose >/=7.8mmol/L: 36/149- 24%) favourable outcome (BI 95-100: 8/36- 22%) versus unfavourable functional outcome (BI <95: 28/36- 78%). Significantly lower odds of favourable functional outcome (BI 95-100) with hyperglycaemia (uOR 0.024, 95%CI 0.01–0.56), p=0.001, but not when adjusting for other factors (aOR 0.081, 95%CI 0.066–.98), p=0.029. No significant difference in glucose in the mortality (N=18/149-12%, Mean 7.02mmol/L, SD 2.21mmol/L) versus survival (N=131/149- 88%, Mean 8.90mmol/L, SD 4.09) group, p=0.084. Significant association between higher glucose and mortality (uOR 1.23, 95%CI 1.05–1.43), p=0.009, but not when adjusting for other factors (aOR 2.13, 95%CI 0.063–7.34), p=0.224. Normoglycaemia patients (Glucose <7.8mmol/L: 133/149- 82%) had a lower proportion of mortality (N=10/113- 9%) versus survival (N= 104/113- 91%) compared with hyperglycaemia (Glucose >/=7.8mmol/L: 36/149- 24%) mortality (N=8/36- 22%) versus survival (N=28/36- 78%). Significantly higher odds of mortality associated with hyperglycaemia (uOR 2.94, 95%CI 1.06–8.16), p=0.032, but not when adjusting for other factors (aOR 1.15, 95%CI 0.095–1.38), p=0.144. | 12.5/14-Low |
| Goyal et al. (2018) | 231/231 (100%) | Ratio/Binary | Hyperglycaemia (>140 mg/dL) versus No hyperglycaemia (</=140 mg/dL) | Significantly less favourable (mRS 0-2: 115/231- 50%) versus unfavourable (mRS 3-6: 116/231- 50%) functional outcome in the hyperglycaemia (>140mg/dL: 88/231- 38%, mRS 0-2: 35/88- 40%, mRS 3-6: 53/88- 60%) versus no hyperglycaemia (</=140mg/dL: 143/231- 62%, mRS 0-2: 80/143- 56%, mRS 3-6: 63/143- 44%) group, p=0.022. Hyperglycaemia was significantly associated with lower odds of favourable (mRS 0-2) functional outcome (univariate OR 0.52, 95%CI 0.030-0.92), p=0.023, (multivariate OR 0.53, 95%CI 0.028-1.02), p=0.059. Significantly higher serum glucose in the unfavourable (mRS 3-6: median 133mg/dL, IQR 107–183mg/dL) versus favourable (mRS 0-2: median 119mg/dL, IQR 101–154mg/dL) functional outcome group, p=0.008. Admission glucose (per 10 mg/dL increase) was not significantly associated with favourable (mRS 0-2) functional outcome (OR 0.095, 95% CI 0.090-1.01), p=0.097, or functional improvement (mRS) (univariate OR 0.096, 95% CI 0.092-1.01), p=0.068. | Shift in ordinal mRS; Mortality and Survival (Binary) | 3 months | Hyperglycaemia was significantly associated with lower odds of functional improvement (shift in mRS) (univariate OR 0.55, 95%CI 0.033-0.92), p=0.021, (multivariate OR 0.53, 95%CI 0.031-0.93), p=0.027. Patients without hyperglycaemia (N=143/231- 62%) had significantly greater functional improvement (mRS) (p=0.018). Significantly greater incidence of mortality (N=59/231- 25%) in the admission hyperglycaemia (glucose >140 mg/dL: 88/231- 38%, mortality: 32/88- 36%) versus no hyperglycaemia (glucose </=140 mg/dL: 143/231- 62%, mortality: 27/143- 19%) group, p=0.005. Hyperglycaemia was a significant predictor of mortality (univariate OR 2.36, 95%CI 1.29-4.33), p=0.006, (multivariate OR 2.76, 95%CI 1.40-5.44),p=0.004. Significantly higher glucose in the mortality (N=59/231- 26%, median 148mg/dL, IQR 112–185mg/dL) versus survival (N=172/231- 74%, median 119mg/dL, IQR 103–155mg/dL) group, p=0.004. Higher glucose (per 10mg/dL increase) was a significant predictor of mortality (univariate OR 1.05, 95%CI 1.01-1.09), p=0.008, (multivariate OR 1.07, 95%CI 1.02-1.12), p=0.004. | 13.5/14-Low |
| Genceviciute et al. (2022) | 1020/1020 (100%) | Ratio/Binary | Hyperglycaemia (≥7.8 mmol/L) versus No hyperglycaemia (<7.8mmol/L) | Lower glucose was a significant predictor of favourable (mRS 0-2) functional outcome (R2 0.0128), p=0.001 (inversely J shaped curve). Significantly greater proportion of patients with a favourable (mRS 0-2: 432/1020- 42%) versus unfavourable (mRS 3-6: 588/1020- 58%) functional outcome did not have hyperglycaemia (<7.8mmol/L: mRS 0-2: 357/738- 50%, mRS 3-6: 381/738- 51%) compared with hyperglycaemia (≥7.8 mmol/L: mRS 0-2: 75/282- 28%, mRS 3-6: 207/282- 73%), P<0.0001. Presence of hyperglycaemia (hyperglycaemia (≥7.8mmol/L) was significantly associated with lower odds of favourable (mRS 0-2) functional outcome when adjusting for age, comorbidities and severity (aOR 00.52, 95%CI 0.37–0.72), P<0.0001. | Excellent (mRS 0-1) and Non-excellent (mRS 2-6) Functional Outcome (Binary); Ordinal mRS; Mortality and Survival (Binary) | 3 months | Significantly less favourable outcome (ordinal mRS) in the hyperglycaemia (≥ 7.8mmol/L: median mRS 4, range 0-6) versus no hyperglycaemia (< 7.8mmol/L: median mRS 2, range 0-6), P<0.0001. Lower glucose was a significant predictor of excellent (mRS 0-1) functional outcome (R^2^.105), p=0.005, (inversely J shaped curve). Significantly fewer patients with an excellent (mRS 0-2) functional outcome had hyperglycaemia (≥7.8mmol/L: 43/282- 16%) versus no hyperglycaemia (<7.8mmol/L: 240/738- 34%), P<0.0001. Hyperglycaemia (≥7.8 mmol/L) was a significant predictor of mortality (aOR 1.8, 95%CI 1.29-20.5), p=0.001. Higher glucose was a significant predictor of Mortality (R^2^ 0.012), p=0.002. | 11/14-Low |
| Rinkel et al. (2020) | 2733/2908 (93.98%) | Ratio/Binary | Hyperglycaemia (≥7.8 mmol/L) versus No hyperglycaemia (<7.8mmol/L) | Significantly lower odds of a favourable (mRS 0-2: 1125/2733- 41%) versus unfavourable (mRS 3-6: 1608/2733- 59%) functional outcome with hyperglycaemia (Glucose ≥7.8 mmol/L, mRS 0-2: 250/841-30%; mRS 3-6: 591/841- 70%) versus no hyperglycaemia (Glucose <7.8mmol/L, mRS 0-2: 875/1892-46%; mRS 3-6: 1017/1892- 54%), ucOR 0.51, 95% CI 0.043–.60; acOR 0.060, 95% CI 0.049–.74. Significant association between higher serum glucose and unfavourable functional outcome (mRS 3-6: aOR 1.12, 95% CI 1.08–1.15). Non-linear association between higher glucose and a shift towards unfavourable functional outcome (mRS 3-6), P<0.001. Glucose <6 mmol/L associated with a shift towards unfavourable functional outcome, but not significantly (aOR 1.16, 95% CI 0.095–1.41). Glucose 6-9 mmol/L significantly associated with shift toward unfavourable functional outcome (aOR 1.27, 95% CI 1.17–1.37). | Mortality and Survival (Binary) | 3 months | Significant association between hyperglycaemia (N=882/2908- 30%) versus no hyperglycaemia (N=439/1892- 23%) and mortality (N=339/841-40, uOR 2.12, 95%CI 1.79–20.51, aOR 1.95, 95%CI 1.60–2.38) (no p-value). | 12/14-Low |
| Bouslama et al. (2018) | 931/931 (100%) | Not Applicable/Binary | Hyperglycaemia (>140 mg/dL) versus No hyperglycaemia (</=140 mg/dL) | Significantly less favourable (mRS 0-2: 431/931- 46%) versus unfavourable (mRS 3-6: 500/931- 54%) functional outcome in the hyperglycaemia (serum glucose >140mg/dL: 289/931- 31%, mRS 0-2: 95/289-33%, mRS 3-6: 194/289 - 67%) versus no hyperglycaemia (glucose </=140mg/dL: 642/931- 69%, mRS 0-2: 336/642- 52%, mRS 3-6: 306/642- 48%) group, P<0.01. Hyperglycaemia (glucose </=140mg/dL: 642/931- 69%) was significantly associated with lower odds of favourable (mRS 0-2) functional outcomes (OR 0.52, 95% CI 0.034), p=0.002. | Mortality and Survival (Binary) | 3 months | Significantly greater mortality (N=210/931- 23%) in the hyperglycaemia (glucose >140mg/dL: 289/931- 31%, mortality: 101/289- 35%) versus no hyperglycaemia (glucose </=140mg/dL: 642/931- 69%, mortality: 109/642- 17%) group, P<0.01. Significant association between hyperglycaemia (glucose >140mg/dL) and mortality (multivariate OR 2.39, 95%CI 1.45-3.82), P<0.001. | 11/14-Low |
| Lasek-Bal et al. (2022) | 417/417 (100%) | Not Applicable/Binary | Hyperglycaemia (>/=140 mg/dL) versus No hyperglycaemia (<140 mg/dL) | Significantly greater hyperglycaemia (blood glucose >140mg/dL: 31/417- 7.43%) in the unfavourable (mRS 3-6: 297/417- 71%, hyperglycaemia: 30/297- 10%) versus favourable (mRS 0-2: 120/417- 29%, hyperglycaemia: 1/120- 0.083%) functional outcome group, p=0.012. However, this did not have sufficient statistical power (Chi^2^ 6.18E–06), p=0.133. | N/A | N/A | N/A | 12.5/14-Low |

AUC=Area Under the Curve; aOR=adjusted odds ratio; BI=Barthel Index; CI=Confidence Interval; DrNI=Dramatic Neurological Improvement; IQR=Inter Quartile Range; mg/dL=milligrams per decilitre; mmol/L=micromoles per Litre; mRS=modified Rankin Score; N/A=Not applicable; NIHSS=National Institute of Health Stroke Scale; OR=Odds Ratio; SD=Standard Deviation; uOR=unadjusted Odds Ratio.

Table S7. Pre-treatment Systolic Blood Pressure (SBP) for Predicting Outcome after Thrombectomy

| **Author(s)** | **Number of Patients (%)** | **Prognostic Factor Continuous**  **Data Type** | **Prognostic Factor Threshold** | **Modified Rankin (mRS) 0-2 versus 3-6 Results** | **Other Outcome Measures(s) (Data type)** | **Other Outcome Measure Timepoint(s)** | **Other Outcome Measure Results** | **Risk of Bias /14** |
| --- | --- | --- | --- | --- | --- | --- | --- | --- |
| Jiang et al. (2015) | 89/89 (100%) | Ratio | N/A | Higher SBP was significantly associated with unfavourable (mRS 3-6: 52/89 - 58%, mean 149.62mmHg, SD 29.19mmHg) versus favourable (mRS 0-2: 37/89 - 42%, mean 137.19mmHg, SD 22.96mmHg) functional outcome (univariate OR 1.02, 95% CI 1-1.03). Insufficient for multivariate analysis. | N/A | N/A | N/A | 12.5/14-Low |
| Chen et al. (2020) | 248/248 (100%) | Ratio | N/A | Significantly greater SBP in the unfavourable (mRS 3-6: 131/248 - 53%, mean 158.4mmHg, SD 23.1mmHg) versus favourable (mRS 0-2: 117/248 - 47%, mean 15.8mmHg, SD 24.1mmHg) functional outcome group, p=0.012. | N/A | N/A | N/A | 13/14-Low |
| Wu et al. (2019) | 118/118 (100%) | Ratio | N/A | No significant difference in SBP in the unfavourable (mRS 3-6: 71/118- 60%, mean 152.4mmHg, SD 25.1mmHg) versus favourable (mRS 0-2: 47/118- 39.8%, mean 153.7mmHg, SD 24.9mmHg) functional outcome group, p=0.94. | N/A | N/A | N/A | 12/14-Low |
| Gordon et al. (2018) | 79/79 (100%) | Ratio | N/A | No significant difference in SBP in the favourable (mRS 0-2: 35/79- 44%, mean 157.4mmHg, SD 31.4mmHg) versus unfavourable (mRS 3-6: 44/79- 56%, mean 1560.5mmHg, SD 25.3mmHg) functional outcome group, p=0.894. | N/A | N/A | N/A | 12/14-Low |
| Sallustio et al. (2019) | 270/270 (100%) | Ratio | N/A | No significant difference in SBP in the favourable (mRS 0-2: 102/270- 38%, mean 146.9mmHg, SD 24.3mmHg) versus unfavourable (mRS 3-6: 168/270- 62%, mean 148.1mmHg, SD 25.8mmHg) functional outcome group, p=0.57. | N/A | N/A | N/A | 12.5/14-Low |
| Yu et al. (2022) | 216/304 (71.05%) | Ratio | N/A | No significant difference in SBP in the favourable (mRS 0-2: 134/216- 62%, mean 148.9mmHg, SD 24.6mmHg) versus unfavourable (mRS 3-6: 82/216- 38%, mean 143.9mmHg, SD 22.3mmHg) functional outcome group, p=0.130. | N/A | N/A | N/A | 11.5/14-Low |
| Lin X et al. (2022a) | 321/312 (102.88%) | Ratio | N/A | No significant difference in SBP in the favourable (mRS 0-2: 133/312- 43%, mean 137.36mmHg, SD 23.36mmHg) versus unfavourable (mRS 3-6: 179/312- 57%, mean 1380.51mmHg, SD 23.21mmHg) functional outcome group, p=0.665. | N/A | N/A | N/A | 13.5/14-Low |
| Zhang et al. (2022) | 258/258 (100%) | Ratio | N/A | No significant difference in SBP in the favourable (mRS 0-2: 106/258- 41%, mean 152.35mmHg, SD 20.99mmHg) versus unfavourable (mRS 3-6: 152/258- 59%, mean 152.49mmHg, SD 25.82mmHg) functional outcome group, p=0.962. | N/A | N/A | N/A | 13/14-Low |
| Zeng et al. (2022) | 110/110 (100%) | Ratio | N/A | No significant difference in SBP in the favourable (mRS 0-2: 49/110- 45%, mean 134.29mmHg, SD 22.65mmHg) versus unfavourable (mRS 3-6: 61/110- 56%, mean 138.44mmHg, SD 21.12mmHg) functional outcome group, p=0.296. | N/A | N/A | N/A | 13/14-Low |
| Lin et al. (2022b) | 84/84 (100%) | Ratio | N/A | No significant difference in SBP in the favourable (mRS 0-2: 42/84- 50%, mean 142.93mmHg, SD 23.87mmHg) versus unfavourable (mRS 3-6: 42/84- 50%, mean 138.86mmHg, SD 25.08mmHg) functional outcome group, p=0.448. | N/A | N/A | N/A | 12.5/14-Low |
| Li et al. (2022) | 329/329 (100%) | Ratio | N/A | No significant difference in SBP in the favourable (mRS 0-2: 137/329- 42%, mean 150.4mmHg, SD 26.1mmHg) versus unfavourable (mRS 3-6: 192/329- 58%, mean 156.2mmHg, SD 26.2mmHg) functional outcome group, p=0.05. No significant association between SBP and unfavourable (mRS 3-6) functional outcome in multivariate analyses adjusting for other variables (aOR's 1.026-1.027, 95%CI's 0.0991-1.012), p's=.636-0.822. | N/A | N/A | N/A | 13/14-Low |
| Diprose et al. (2020) | 432/432 (100%) | Ratio | N/A | Higher SBP in the unfavourable (mRS 3-6: 190/432 - 44%, mean 154mmHg, SD 28mmHg) versus favourable (mRS 0-2: 242/432 - 56%, mean 152mmHg, SD 27mmHg) functional outcome group (no statistical comparison). | N/A | N/A | N/A | 12/14-Low |
| Cho et al. (2019) | 378/378 (100%) | Ratio | N/A | Significantly higher SBP in the unfavourable (mRS 3-6: 229/378- 61%, mean 139.0mmHg, SD 23.6mmHg) versus favourable (mRS 0-2: 149/378- 39%, mean 133.6mmHg, SD 21.3mmHg) functional outcome group, p=0.023. No significant difference in SBP initial (immediately before thrombectomy) in the favourable (mRS 0-2: 149/378- 39% mean 137.2mmHg, SD 2.4mmHg) versus unfavourable (mRS 3-6: 229/378- 61%, mean 139.7mmHg, SD 24.4mmHg) functional outcome group, p=0.286. No significant association between SBP initial and favourable (mRS 0-2) functional outcome (univariate OR 0.095, 95%CI 0.087-1.04), p=0.30, interaction p=0.43, or any change in mRS distribution (1 point increments-ordinal) (univariate OR 1.03, 95%CI 0.096-1.12), p=0.41, interaction p= 0.043. | Mortality and Survival (Binary) | 3 months | No significant difference in SBP initial in the survival (mean 138.8mmHg, SD 220.5mmHg) versus mortality (mean 138.8mmHg, SD 270.5mmHg) group, p=0.89. No significant association between SBP initial and mortality (multivariate OR 1, 95%CI 0.098-1.02), p=0.93, interaction p=0.37. | 14/14-Low |
| Sun et al. (2021) | 212/212 (100%) | Ratio | N/A | Significantly greater SBP in the unfavourable (mRS 3-6: 98/212 - 46%, mean 145.34mmHg, SD 23.34mmHg) versus favourable (mRS 0-2: 114/212 - 54%, mean 137.68mmHg, SD 23.95mmHg) functional outcome group, p=0.02. No significant association between SBP and favourable (mRS 0-2) functional outcome (OR 1.01, 95%CI 0.099-1.02), p=0.513. | Mortality and Survival (Binary) | 3 months | No significant difference in SBP in the mortality (N= 31/212- 14.6%, mean 147.90mmHg, SD 26.22mmHg) versus survival (N=181/212- 85%, mean 14.05mmHg, SD 23.39mmHg) group, p=0.091. | 9.5/14-Moderate |
| Anadani et al. (2019) | 1149/1245 (92.29%) | Ratio | N/A | No significant difference in SBP in the unfavourable (mRS 3-6: 582/1149- 51%, mean 145mmHg, SD 28mmHg) versus favourable (mRS 0-2: 567/1149- 49%, mean 144mmHg, SD 27mmHg) functional outcome group, p=0.77. | Mortality and Survival (Binary) | 3 months | No significant difference in SBP in the Survival (N=946/1149- 82.3%, mean 144mmHg, SD 27mmHg) versus Mortality (N=203/1149- 17.6%, mean 145mmHg, SD 30mmHg) groups, p=0.6. | 13/14-Low |
| Goyal et al. (2017) | 116/116 (100%) | Ratio | N/A | Significantly lower SBP in patients with favourable (mRS 0-2: 38/116- 32.7%, mean 151mmHg, SD 24mmHg) versus unfavourable (mRS 3-6: 78/116 - 67%, mean 165mmHg, SD 28mmHg) functional outcome, p= 0.001. 10mmHg incremental increase in admission SBP was independently associated with a lower likelihood of favourable functional outcomes (mRS 0-2: Coefficient -0.452, SE -0.180, aOR 0.064, 95% CI 0.045-0.91), p=0.012. | Mortality and Survival (Binary); Infarct Volume (Ratio) | In-Hospital (FIV and Mortality) and 3 months (Mortality) | Significantly higher mean admission SBP in the in-hospital mortality (169mmHg +/-34mmHg) versus survival (156mmHg +/-24mmHg) group, p=0.021, but not for 3-month mortality (116mmHg +/-35mmHg) versus survival (156mmHg +/-23mmHg) group, p=0.16. Higher pre-treatment SBP was positively correlated with increased FIV (r: +.225; p=0.020). A 10mmHg incremental increase in pre-treatment SBP was independently associated with an increase of 12cm3 in FIV (standardised linear regression coefficient 0.0415, unstandardised coefficient 12cm3, 95% CI 3-21, p=0.010) after adjusting for multiple factors. | 13/14-Low |

CI=Confidence interval; DBP=Diastolic Blood Pressure; FIV=Final Infarct Volume; mmHg=millimetres of mercury; N/A=Not applicable; OR=Odds Ratio; SBP=Systolic Blood Pressure; SD=Standard Deviation.

Table S8. Pre-treatment Diastolic Blood Pressure (DBP) for Predicting Outcome after Thrombectomy

| **Author(s)** | **Number of Patients (%)** | **Prognostic Factor Continuous Data Type** | **Prognostic Factor Threshold** | **Modified Rankin (mRS) 0-2 versus 3-6 Results** | **Other Outcome Measures(s) (Data type)** | **Other Outcome Measure Timepoint(s)** | **Other Outcome Measure Results** | **Risk of Bias /14** |
| --- | --- | --- | --- | --- | --- | --- | --- | --- |
| Chen et al. (2020) | 248/248 (100%) | Ratio | N/A | No significant difference in DBP in the unfavourable (mRS 3-6: 131/248 - 53%, mean 80.7mmHg, SD 12.6mmHg) versus favourable (mRS 0-2: 117/248 - 47%, mean 78.9mmHg, SD 13.2mmHg) functional outcome group, p=0.289. | N/A | N/A | N/A | 13/14-Low |
| Wu et al. (2019) | 118/118 (100%) | Ratio | N/A | No significant difference in DBP in the unfavourable (mRS 3-6: 71/118- 60%, mean 84.9mmHg, SD 15mmHg) versus favourable (mRS 0-2: 47/118- 39.8%, mean 88.8mmHg, SD 15.7mmHg) functional outcome group, p=0.1871. | N/A | N/A | N/A | 12/14-Low |
| Yu et al. (2022) | 304/216 (71.05%) | Ratio | N/A | No significant difference in DBP in the favourable (mRS 0-2: 134/216- 62%, mean 86.4mmHg, SD 18.2mmHg) versus unfavourable (mRS 3-6: 82/216- 38%, mean 84.7mmHg, SD 13.1mmHg) functional outcome group, p=0.437. | N/A | N/A | N/A | 11.5/14-Low |
| Lin X et al. (2022a) | 312/312 (100%) | Ratio | N/A | No significant difference in DBP in the favourable (mRS 0-2: 133/312- 43%, mean 83.09mmHg, SD 14.44mmHg) versus unfavourable (mRS 3-6: 179/312- 57%, mean 84.7mmHg, SD 15.42mmHg) functional outcome group, p=0.348. | N/A | N/A | N/A | 13.5/14-Low |
| Zhang et al. (2022) | 258/258 (100%) | Ratio | N/A | No significant difference in DBP in the favourable (mRS 0-2: 106/258- 41%, mean 85.64mmHg, SD 15.91mmHg) versus unfavourable (mRS 3-6: 152/258- 59%, mean 85.7mmHg, SD 14.64mmHg) functional outcome group, p=0.977. | N/A | N/A | N/A | 13/14-Low |
| Zeng et al. (2022) | 110/110 (100%) | Ratio | N/A | No significant difference in DBP in the favourable (mRS 0-2: 49/110- 45%, mean 79.29mmHg, SD 16.07mmHg) versus unfavourable (mRS 3-6: 61/110- 56%, mean 80.95mmHg, SD 12.25mmHg) functional outcome group, p=0.284. | N/A | N/A | N/A | 13/14-Low |
| Lin et al. (2022b) | 84/84 (100%) | Ratio | N/A | No significant difference in DBP in the favourable (mRS 0-2: 42/84- 50%, mean 82.43mmHg, SD 15.69mmHg) versus unfavourable (mRS 3-6: 42/84- 50%, mean 82.9mmHg, SD 15.18mmHg) functional outcome group, p=0.888. | N/A | N/A | N/A | 12.5/14-Low |
| Li et al. (2022) | 329/329 (100%) | Ratio | N/A | No significant difference in DBP in the favourable (mRS 0-2: 137/329- 42%, mean 88.7mmHg, SD 17.4mmHg) versus unfavourable (mRS 3-6: 192/329- 58%, mean 89.4mmHg, SD 15.3mmHg), p=0.701. | N/A | N/A | N/A | 13/14-Low |
| Jiang et al. (2015) | 89/89 (100%) | Ratio | N/A | Greater DBP in the unfavourable (mRS 3-6: 47/89 -53%, mean 86.13mmHg, SD 14.2mmHg) versus favourable (mRS 0-2: 37/89 - 410.57%, mean 80.19mmHg, SD 12.28mmHg) functional outcome group (univariate OR 1.03, 95%CI 1-1.07). No p-value but unlikely significant. Insufficient for multivariate analysis. | Mortality and Survival (Binary) | 3 months | N/A | 12.5/14-Low |
| Cho et al. (2019) | 378/378 (100%) | Ratio | N/A | No significant difference in DBP in the favourable (mRS 0-2: 149/378- 39.4%, mean 83.6mmHg, SD 13.1mmHg) versus unfavourable (mRS 3-6: 229/378- 61%, mean 86.3mmHg, SD 15.7mmHg) functional outcome group, p=0.075, or initial DBP in the favourable (mRS 0-2: 149/378- 39.4%, mean 76.3mmHg, SD 12.4mmHg) versus unfavourable (mRS 3-6: 229/378- 60.5%, mean 77.3mmHg, SD 14mmHg) functional outcome group, p=0.464. No significant association between initial DBP and favourable (mRS 0-2) functional outcome (univariate OR 0.094, 95%CI 0.081-1.1), p=0.46, interaction p=0.16. | Mortality and Survival (Binary) | 3 months | No significant association between DBP initial and mortality (mean 82mmHg, SD 17mmHg) versus survival (mean 76.4mmHg, SD 12.9mmHg), p=0.08. | 14/14-Low |
| Sun et al. (2021) | 212/212 (100%) | Ratio | N/A | No significant difference in DBP in the favourable (mRS 0-2: 114/212 - 54%, mean 85.71mmHg, SD 14.79mmHg) versus unfavourable (mRS 3-6: 98/212 - 46%, mean 87.44mmHg, SD 15.67mmHg) functional outcome group, p=0.41. | Mortality and Survival (Binary) | 3 months | No significant difference in DBP in the mortality (N=31/212 - 15%, mean 85.84mmHg, SD 15.85mmHg) versus survival (N=181/212- 85%, mean 86.62mmHg, SD 15.11mmHg) group, p=0.791. | 9.5/14-Moderate |
| Anadani et al. (2019) | 1245/1057 (84.9%) | Ratio | N/A | No significant difference in DBP in the unfavourable (mRS 3-6: 582/1057- 55%, mean 79mmHg, SD 20mmHg) versus favourable (mRS 0-2: 567/1057- 49%, mean 81mmHg, SD 19mmHg) functional outcome group, p=0.09. | Mortality and Survival (Binary) | 3 months | No significant difference in DBP in the mortality (N=203/1149- 17.6%, mean 81mmHg, SD 18mmHg) versus survival (N=946/1149-82.3%, mean 77mmHg, SD 21mmHg) group, p=0.065. | 13/14-Low |
| Goyal et al. (2017) | 116/116 (100%) | Ratio | N/A | No significant difference in DBP in the favourable (mRS 0-2: 38/116- 32.7%, mean 88mmHg, SD 20mmHg) versus unfavourable (mRS 3-6: 78/116- 67%, mean 89mmHg, SD 22mmHg) functional outcome group, p=0.858. | Mortality and Survival (Binary); Infarct Volume (Ratio) | In-Hospital (FIV and Mortality) and 3 months (Mortality) | No significant difference in DBP in the in-hospital mortality (mean 94mmHg, SD 24mmHg) versus survival (mean 86mmHg, SD 18mmHg) group, p=0.08. No significant difference in DBP in the 3-month mortality (mean 91mmHg, SD 24mmHg) versus survival (mean 86mmHg, SD 18mmHg) group, p=0.315. DBP selected for inclusion in multivariate model (P<0.1 on linear regression) but was not independently associated with FIV. | 13/14-Low |

BP=Blood Pressure; DBP=Diastolic Blood Pressure; FIV=Final Infarct Volume; mmHg=millimetres of mercury; NA=Not Applicable; SBP=Systolic Blood Pressure; SD=Standard Deviation

Table S9. Pre-treatment Electrocardiogram (ECG) detected Atrial Fibrillation (AF) for Predicting Outcome after Thrombectomy

| **Author(s)** | **Number of Patients (%)** | **Prognostic Factor Categorical Data Type and Threshold** | **Modified Rankin (mRS) 0-2 versus 3-6 Results** | **Other Outcome** | **Other Outcome Time Period** | **Other Outcome Results** | **Risk of Bias /14** |
| --- | --- | --- | --- | --- | --- | --- | --- |
| Pinho et al. (2021) | 489/489 (100%) | Binary-Present/  Absent | Significantly greater incidence of ECG detected AF (N=253/489-47%) in the unfavourable (mRS 3-6: 183/327- 56%) versus favourable (mRS 0-2: 70/162- 43.2%) functional outcome group, p=0.008. | N/A | N/A | N/A | 13.5/14-Low |
| Nisar et al. (2021) | 188/188 (100%) | Binary-Present/  Absent | No significant difference in ECG detected AF (N=83/188-44%) in the favourable (mRS 0-2: 22/54- 4.75%) versus unfavourable (mRS 3-6: 61/134- 450.53%) functional outcome group (95% CI 0.064–2.31), p=0.55. | Mortality and Survival (Binary) | 3 months | No significant difference in acute AF (N=83/188- 44%) in the mortality (N=22/58- 38%) versus survival (N=61/130-47%) group (95% CI, 0.037–1.30), p=0.251. | 12.5/14-Low |
| Soize et al. (2013) | 59/59 (100%) | Binary-Present/  Absent | No significant difference in ECG detected AF (N=16/59- 27%) in the favourable (mRS 0-2: 9/34-260.5%) versus unfavourable (mRS 3-6: 7/25- 28%) functional outcome group, p=0.89. | Mortality and Survival (Binary) | 3 months | No significant difference in acute AF (N=16/59- 27%) in the mortality (N=3/12- 25%) versus survival (N=13/47- 28%) group, p=0.85. | 12/14-Low |

AF=Atrial Fibrillation; CI=Confidence Interval; ECG=Electrocardiogram; N/A=Not Applicable

Table S10. Pre-treatment Consciousness measured by Glasgow Coma Scale (GCS) for Predicting Outcome after Thrombectomy

| **Author(s)** | **Number of Patients (%)** | **Prognostic Factor Continuous Data Type** | **Modified Rankin (mRS) 0-2 versus 3-6 Results** | **Other Outcome Measures(s) (Data type)** | **Other Outcome Measure Timepoint(s)** | **Other Outcome Measure Results** | **Risk of Bias /14** |
| --- | --- | --- | --- | --- | --- | --- | --- |
| Costalat et al. (2012) | 50/50 (100%) | Ordinal (treat as ratio) | No significant difference, with a trend toward higher GCS (greater consciousness), in the favourable (mRS 0-2: 26/50- 52%, mean 13.1, SD 3.2, range 3-15) versus unfavourable (mRS 3-6: 24/50 - 48%, mean 11, SD 4.9, range 3-15) functional outcome group, p=0.053. | N/A | N/A | N/A | 11.5/14-Low |
| Phuong et al. (2022a) | 49/49 (100%) | Ordinal (treat as ratio) | No significant difference in GCS in the favourable (mRS 0-2: 22/49- 45%, mean score 12, SD 2.6) versus unfavourable (mRS 3-6: 27/49- 55%, mean score 9, SD 2.9) functional outcome group, P>0.05. | N/A | N/A | N/A | 10/14-Moderate |

GCS=Glasgow Coma Scale/Score; SD=Standard Deviation; N/A=Not Applicable

Funnel Plots


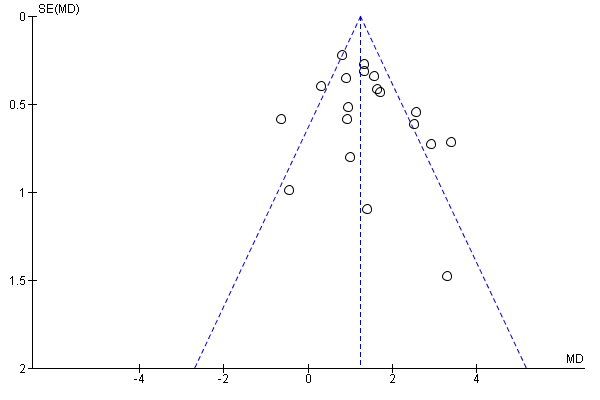


Figure S1. Glucose (mmol/L; continuous) Funnel Plot

**
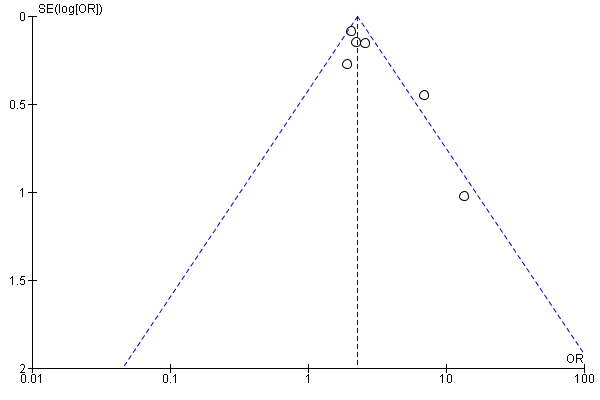
**

Figure S2. Hyperglycaemia (Glucose >/=7.8mmol/l; Binary) Funnel Plot


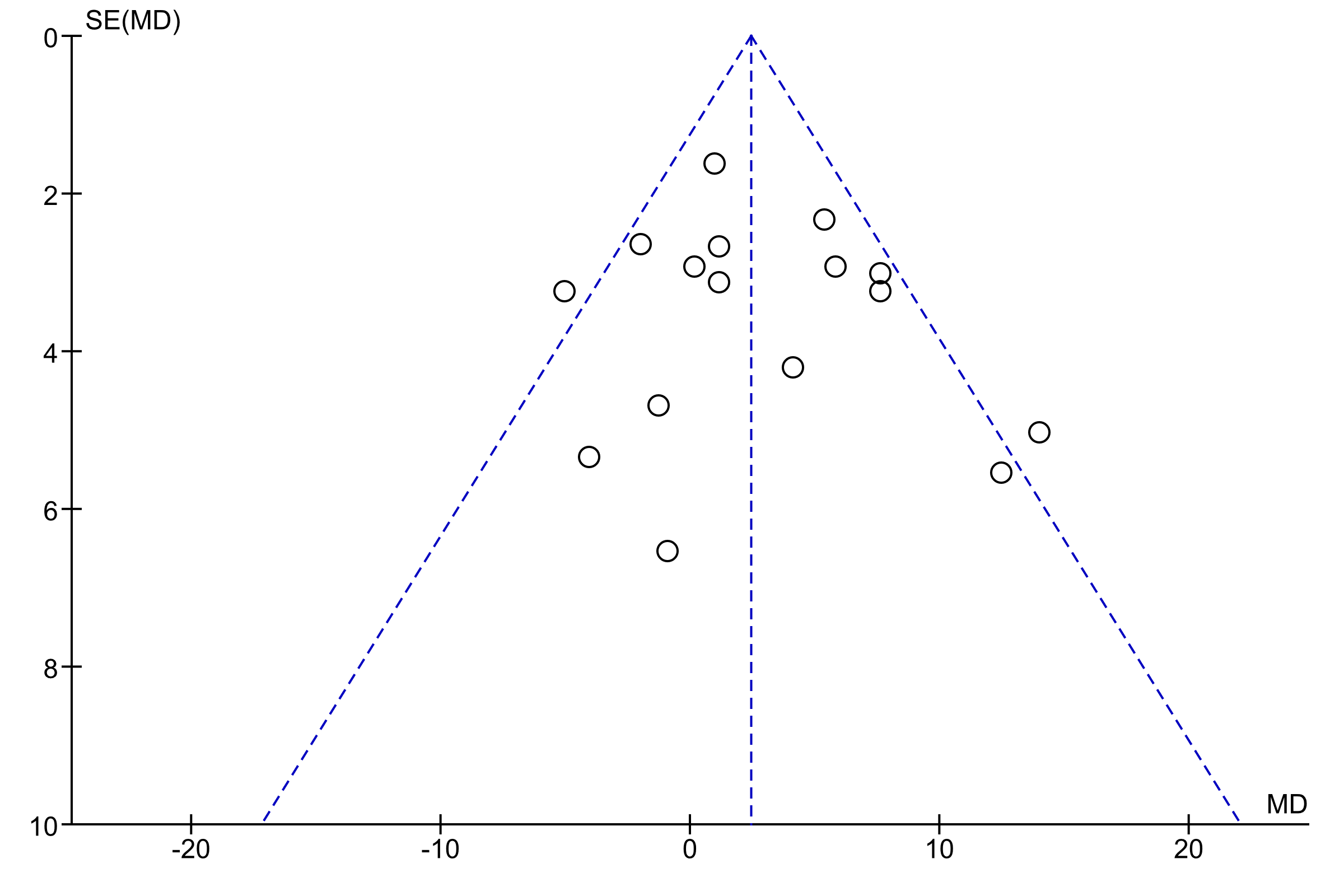


Figure S3. Systolic Blood Pressure (mmHg; continuous) Funnel Plot


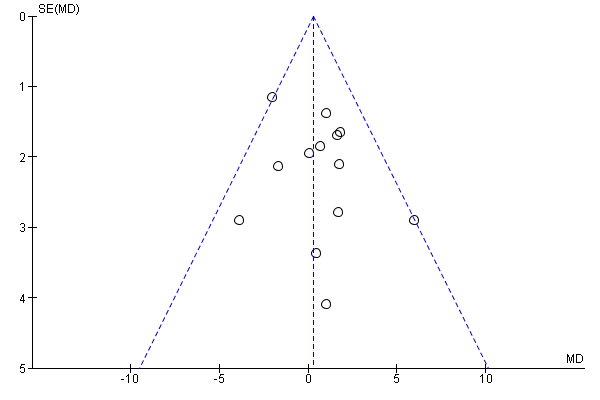


Figure S4. Diastolic Blood Pressure (mmHg; continuous) Funnel Plot


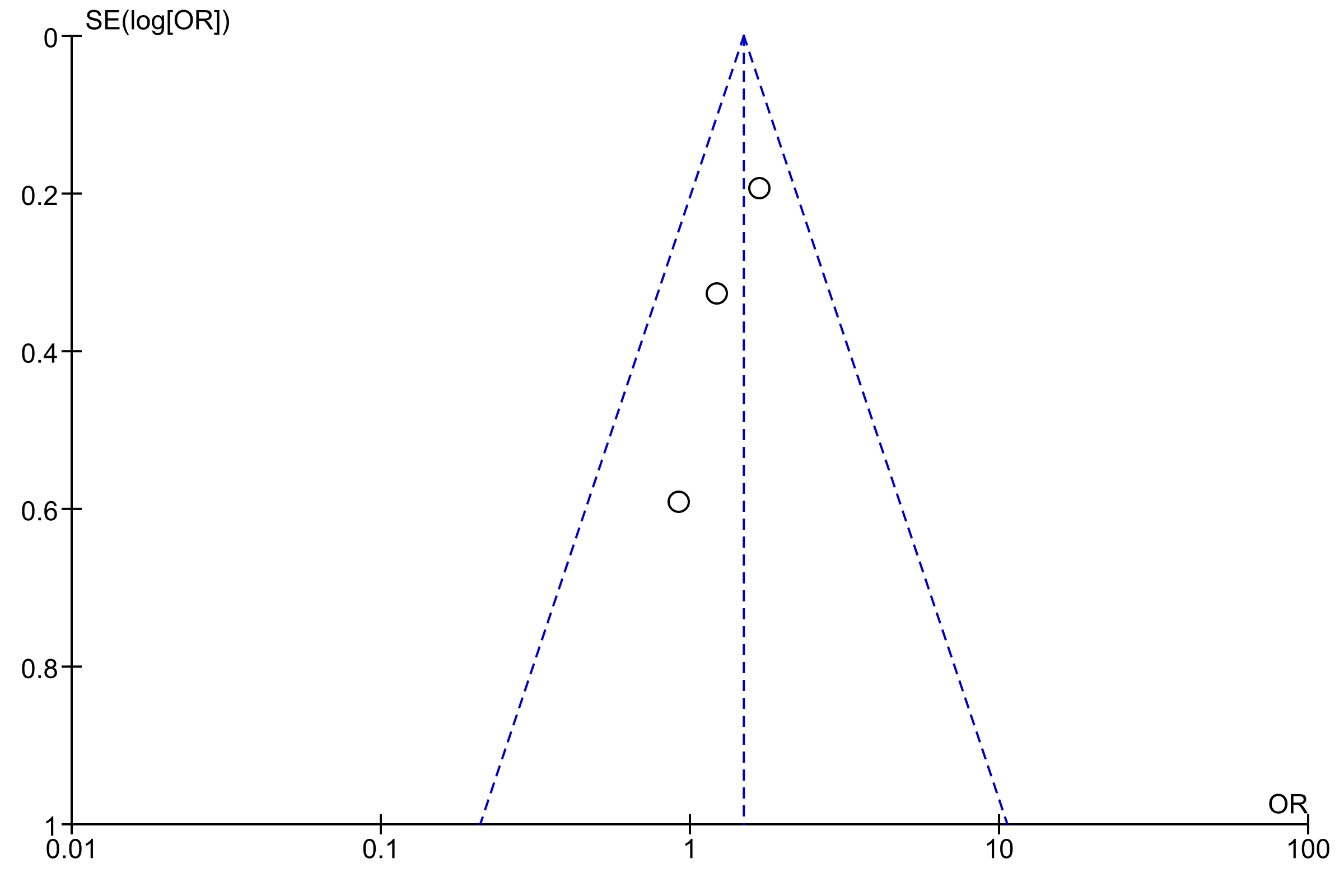


Figure S5. Atrial Fibrillation (detected on ECG; Binary) Funnel Plot


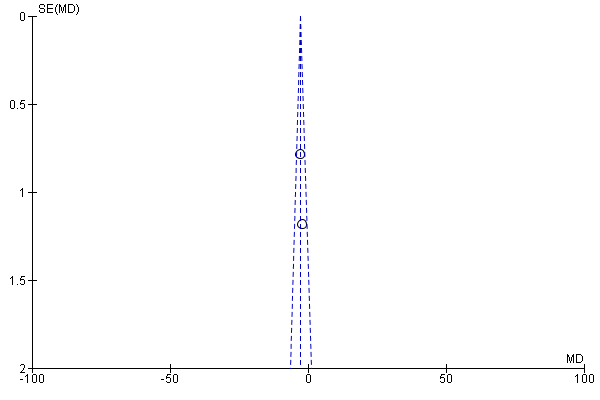


Figure S6. Conscious level (measured by Glasgow Coma Scale; continuous) Funnel Plot
